# Genome-Wide Sequencing Reveals Two Major Sub-Lineages in the Genetically Monomorphic Pathogen *Xanthomonas Campestris* Pathovar *Musacearum*

**DOI:** 10.3390/genes3030361

**Published:** 2012-07-04

**Authors:** Arthur Wasukira, Johnbosco Tayebwa, Richard Thwaites, Konrad Paszkiewicz, Valente Aritua, Jerome Kubiriba, Julian Smith, Murray Grant, David J. Studholme

**Affiliations:** 1 Biosciences, University of Exeter, Geoffrey Pope Building, Stocker Road, Exeter EX4 4QD, UK; E-Mails: awasukira@gmail.com (A.W.); tayebwajb@gmail.com (J.T.); k.h.paszkiewicz@exeter.ac.uk (K.P.); m.r.grant@exeter.ac.uk (M.G.); 2 National Crops Resources Research Institute (NaCRRI), Kampala 7084, Uganda; E-Mails: arituavalentine@yahoo.com (V.A.); jkubiriba@kari.go.ug (J.K.); 3 The Food and Environment Research Agency, Sand Hutton, York YO41 1LZ, UK; E-Mails: richard.thwaites@fera.gsi.gov.uk (R.T.); julian.smith@fera.gsi.gov.uk (J.S.)

**Keywords:** banana *Xanthomonas* wilt, whole-genome sequencing, SNP, molecular markers, *Ensete ventricosum*, *Xanthomonas vasicola*

## Abstract

The bacterium *Xanthomonas campestris* pathovar *musacearum* (*Xcm*) is the causal agent of banana *Xanthomonas* wilt (BXW). This disease has devastated economies based on banana and plantain crops (*Musa* species) in East Africa. Here we use genome-wide sequencing to discover a set of single-nucleotide polymorphisms (SNPs) among East African isolates of *Xcm*. These SNPs have potential as molecular markers for phylogeographic studies of the epidemiology and spread of the pathogen. Our analysis reveals two major sub-lineages of the pathogen, suggesting that the current outbreaks of BXW on *Musa* species in the region may have more than one introductory event, perhaps from Ethiopia. Also, based on comparisons of genome-wide sequence data from multiple isolates of *Xcm* and multiple strains of *X. vasicola* pathovar *vasculorum*, we identify genes specific to *Xcm* that could be used to specifically detect *Xcm* by PCR-based methods.

## 1. Introduction

The bacterium *Xanthomonas campestris* pathovar *musacearum* (*Xcm*) is the causal agent of banana *Xanthomonas* wilt (BXW). This disease has devastated economies based on banana and plantain crops (*Musa* species) in East Africa [1]. *Xcm* was first described as a wilt-causing pathogen on enset (*Ensente ventricosum*), a plant closely related to banana that is a staple crop in the highlands of Ethiopia [2]. In 1974 Yirgou and Bradbury [3] wrote that “*Great care should be taken to see that enset wilt does not escape and establish itself on banana in other parts of the world where it could pose a serious problem on this crop*”. Ominously, one and a half decades later, a major epidemic of this disease was reported in Uganda [4]. Subsequently it has spread into many banana-growing regions around the Great Lakes in Uganda, Kenya, Tanzania, Democratic Republic of Congo, Rwanda and Burundi [4,5,6,7]. Efforts are underway to tackle this pathogen by a number of different measures including cultural practices [8] and genetic modification of the crop [9,10].

Although currently classified as a member of the species *Xanthomonas campestris*, we recently showed that *Xcm* is more likely to be a strain of the species *Xanthomonas vasicola* [11]. We previously [12] generated complete genome sequences for a single isolate of *Xcm* from banana in Uganda (NCPPB4381) and for a single isolate of *X. vasicola* pathovar *vasculorum* (*Xvv*) that is non-pathogenic on banana and was isolated from sugarcane in Zimbabwe (NCPPB702). These two isolates share identical gyrase B DNA sequences, consistent with their very close evolutionary relationship [11,13].

Differences between these two genome sequences revealed several candidate genes that might play a role in adaptation to the banana host. These may also be useful tools in identifying genes for deployment of disease resistance. Specifically, these included homologues of effectors secreted and translocated by the type III secretion system (T3SS). T3SS effectors have previously been shown to contribute to host-specificity acting as virulence and/or avirulence factors [14]. In common with most previously sequenced *Xanthomonas* genomes, *Xcm* encodes homologues of the effectors AvrBs2, AvrGf1, XopF, XopK, XopL, XopN, XopP, XopQ, XopR, XopX and XopZ as well as homologues of XopA, XopB, XopG, XopH, XopI, XopY, XopAA, XopAD, XopAE and XopAK, which are found in some other *Xanthomonas* species [12,14]. *Xcm* also encodes homologues of *P. syringae* effectors HopW1 and HopAF1 and *Ralstonia solanacearum* putative effector RipT [12]. *Xcm* encodes two predicted YopJ-like C55 cysteine proteases (RefSeq accessions ZP_06491730 and ZP_06492219) that are absent from *Xvv* 702. On the other hand, *Xvv* 702 encodes a close homologue (ZP_06483517) of XopAF (also known as AvrXv3), which is absent from *Xcm* [12].

Previous work showed that *Xcm* is a highly monomorphic pathogen and no specific genetic differences have yet been detected among different isolates using traditional typing and diagnostic methods [13,15]. Affordable complete genome sequencing now makes it feasible to identify cryptic genetic diversity among isolates of a genetically monomorphic pathogen [16], though this approach is only just starting to be applied to monomorphic phytopathogens [17].

Here we use genome-wide sequencing to discover a set of single-nucleotide polymorphisms (SNPs) among East African isolates of *Xcm*. These SNPs have potential as molecular markers for phylogeographic studies of the epidemiology and spread of the pathogen. Our analysis reveals the presence of at least two major sub-lineages of the pathogen; *Xcm* isolates from Uganda, Kenya, Tanzania and Burundi are genetically distinct from isolates collected in Ethiopia, DR Congo and Rwanda, suggesting that the current outbreaks of BXW on *Musa* species in the region may have more than one introduction.

## 2. Results and Discussion

### 2.1. Genome Sequencing

We used the Illumina GA2x sequencing platform to generate genome-wide sequence data for 13 isolates of *Xcm* available from the National Collection of Plant Pathogenic Bacteria (NCPPB). We also sequenced a further three African isolates of *Xvv*, a pathovar that is very closely related to *Xcm* but non-pathogenic on banana. We also included in our analyses the genome sequence data from *Xcm* NCPPB4381 and *Xvv* NCPPB702 that we published previously [12]. Table 1 lists the sequenced isolates and the depth to which each was sequenced. We submitted all raw sequence data to the Sequence Read Archive [18]. Note that another isolate from Tanzania, NCPPB4393, is described in the NCPPB’s catalogue as *Xcm*, but we previously sequenced its genome and showed that it is actually *Xanthomonas sacchari* [19]. We generated genome assemblies *de novo* for NCPPB2005, NCPPB4379, NCPPB4380, NCPPB4384, NCPPB4392, NCPPB4394, NCPPB1326, NCPPB1381 and NCPPB206 using Velvet 1.1.04 [20]. These have been submitted to GenBank [21] with accession numbers AKBE00000000, AKBF00000000, AKBG00000000, AKBH00000000, AKBI00000000, 

**Table 1:** Isolates of *X. campestr* is pv. *musacearum* (*Xcm)* and *X. vasicola* pv. *vasculorum* (*Xvv)* subjected to genome-wide sequencing. All *Xcm* isolates were originally collected from diseased banana plants except for NCPPB2005, which was isolated from *Ensete ventricosum*. All *Xvv* isolates were originally collected from sugarcane, except for NCPPB206, which was isolated from maize.

Isolate	Source and Date of Isolation		Coverage	SRA Accession
*Xcm* NCPPB2005	Ethiopia 1967		72×	SRR489154.7
*Xcm* NCPPB2251	Ethiopia 1969		13×	SRR494492.2
*Xcm* NCPPB4379	Uganda (Kayunga) 2007		102×	SRR494484.2
*Xcm* NCPPB4380	Uganda (Kiboga) 2007		113×	SRR494485.2
*Xcm* NCPPB4381	Uganda (Luwero) 2007		56×	SRR020203.3
*Xcm* NCPPB4383	Uganda (Wakiso) 2007		11×	SRR494493.2
*Xcm* NCPPB4384	Uganda (Nakaongola) 2007		55×	SRR494488.2
*Xcm* NCPPB4387	D. R. Congo (Kivu province) 2007		13×	SRR494494.1
*Xcm* NCPPB4389	Rwanda (Gisenyi province) 2007		16×	SRR494495.2
*Xcm* NCPPB4392	Tanzania (Muleba district, Kagera region) 2007		72×	SRR494498.3
*Xcm* NCPPB4394	Tanzania (Muleba district, Kagera region) 2007		92×	SRR494489.1
*Xcm* NCPPB4395	Tanzania (Muleba district, Kagera region) 2007		117×	SRR494490.2
*Xcm* NCPPB4433	Burundi 2008		13×	SRR494496.1
*Xcm* NCPPB4434		Kenya (Teso district) 2008	15×	SRR494497.1
*Xvv* NCPPB206		South Africa 1948	70×	SRR494500.3
*Xvv* NCPPB702		Zimbabwe 1959	35×	SRR020202.3
*Xvv* NCPPB1326		Zimbabwe 1962	63×	SRR494491.5
*Xvv* NCPPB1381		Zimbabwe 1962	66×	SRR494499.3

AKBJ00000000, AKBK00000000, AKBL00000000 and AKBM00000000. The most contiguous of these assemblies was for NCPPB4384. This consisted of 84 scaffolds, of which the 12 longest scaffolds were each at least 154 Kb long and accounted for more than 2.5 Mb; that is the N_50_ length was 154 Kb for NCPPB4384. The N_50_ lengths for the other *Xcm* assemblies were 56 Kb (NCPPB2005), 146 Kb (NCPPB4379), 147 Kb (NCPPB4380), 87 Kb (NCPPB4392) and 151 Kb (NCPPB4394).

### 2.2. Distinguishing *Xcm* from *Xvv*

The currently used method for identifying BXW is by isolation of bacteria from the infected plant and performing fatty acid and metabolic analyses [4]. However, this approach is only appropriate once symptoms become visible, by which time it may be too late to control or eradicate the pathogen. An alternative approach, amenable to the rapid detection and identification of bacterial plant pathogens is the use of the polymerase chain reaction (PCR). A specific assay for detecting *Xcm* has recently been proposed based on PCR amplification of the *hrpB* gene [22]. However, this gene is also conserved in *Xvv* and this assay was unable to distinguish between *Xcm* and non-banana-pathogenic isolates *Xvv* NCPPB702 and NCPPB1326 [22]. Another study [23] generated several PCR primer pairs that were highly specific for *Xcm* but this study did not utilize *Xcm* or *Xvv* genomic sequence but rather used sequences from a range of other xanthomonads and so the candidate primers had to be tested for specificity by trial and error. Another recent study [24] exploited our previous [12] *Xcm* and *Xvv* draft genome sequence data to rationally design primers specific for *Xcm*. However, this was based on genome sequence from a single isolate of *Xvv* and a single isolate of *Xcm*. Until now, little was known about sequence diversity among isolates. Therefore, we identified a set of genes that are conserved in all of the sequenced isolates of *Xcm* but are absent from all the sequenced isolates of *Xvv* and are therefore candidates for use in an *Xcm*-specific PCR assay. Examples of these genes are listed in Table 2. Note that this list of genes was not generated by aligning assembled genome sequences. We aligned raw unassembled sequence reads against the previously published *Xcm* NCPPB4381 reference genome sequence [12] using the Burrows-Wheeler Aligner BWA [25]. This approach has the advantage of avoiding assembly artifacts and problems arising from incomplete assemblies. In our BWA alignments of raw Illumina sequence reads *versus* the reference genome sequence, the full length of each gene was covered by reads (depth of one or greater) from all our *Xcm* Illumina sequence datasets. Each of these genes has no matches (*i.e.*, zero depth of) to any sequence reads in our *Xvv* Illumina datatsets (as judged from the BWA alignments).

**Table 2:** Candidate genes for development of an *Xcm*-specific PCR-based assay. The listed genes conserved in all of the sequenced *Xcm* isolates but absent from all of the sequenced *Xvv* isolates. Presence or absence of each gene was assessed based on alignment of Illumina sequence reads from each isolate against the *Xcm* NCPPB4381 reference genome sequence (RefSeq: ACHT00000000) using BWA [25].

RefSeq Locus tag	Predicted Gene Product
XcampmN_010100002667	hypothetical protein
XcampmN_010100009057	general secretion pathway protein D
XcampmN_010100016989	transposase
XcampmN_010100016984	phage-related integrase
XcampmN_010100014552	hypothetical protein
XcampmN_010100013878	DNA-cytosine methyltransferase
XcampmN_010100013483	hypothetical protein
XcampmN_010100011643	conjugal transfer relaxosome component TraJ
XcampmN_010100011578	hypothetical protein
XcampmN_010100011573	Fis family transcriptional regulator
XcampmN_010100011558	hypothetical protein
XcampmN_010100011553	hypothetical protein
XcampmN_010100010854	hypothetical protein
XcampmN_010100010849	XRE family transcriptional regulator
XcampmN_010100006985	hypothetical protein
XcampmN_010100004971	exported protein
XcampmN_010100004961	virulence regulator
XcampmN_010100004956	hypothetical protein
XcampmN_010100004736	hypothetical protein
XcampmN_010100001342	ISXo2 putative transposase
XcampmN_010100001332	ABC-type antimicrobial peptide transport system ATPase component
XcampmN_010100001327	RND family efflux transporter MFP subunit
XcampmN_010100013888	ISxac1 transposase
XcampmN_010100011563	putative DNA methylase
XcampmN_010100004966	integrase
XcampmN_010100001337	peptide ABC transporter permease
XcampmN_010100013883	restriction endonuclease-like protein
XcampmN_010100000225	putative secreted protein
XcampmN_010100000622	fimbrillin
XcampmN_010100015677	methyltransferase
XcampmN_010100016677	Putative acetylhydrolase

### 2.3. The Sequenced *Xcm* Isolates Comprise a Single Monophyletic Clade

**Figure 1:**
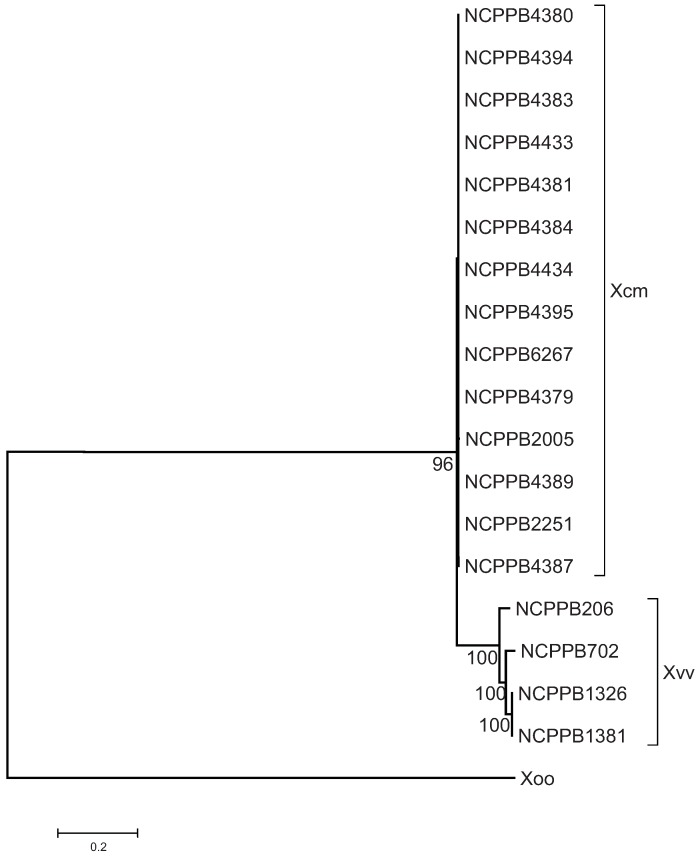
East African isolates of *Xanthomonas campestris* pv. *musacearum* (*Xcm*) from banana and enset comprise a monophletic clade closely related to *Xanthomonas vasicola*. A maximum likelihood phylogenetic tree was generated as described in the Experimental Section. The tree was rooted with *X. oryzae* pv. *oryzae* MAFF 311018 (“Xoo”) as the outgroup. The tree is based on 21,525 SNPs in 19 taxa. Branch lengths are drawn to scale and measured in the number of substitutions per site. Bootstrap values are given as percentages from 500 bootstrap trials.

We identified SNPs among the *Xcm* and *Xvv* isolates based on BWA [25] alignments of our Illumina sequence data against the *X. oryzae* pv. *oryzae* MAFF 311018 reference genome sequence (RefSeq: NC_007705). Based on nucleotides found at 21,525 polymorphic positions we generated the maximum likelihood phylogenetic tree shown in Figure 1. This clearly groups all of the sequenced *Xcm* isolates into a single distinct clade that is closely related to but distinct from the sequenced *Xvv* isolates.

### 2.4. *Xcm* Isolates from Uganda, Kenya, Tanzania and Burundi are Genetically Distinct from Isolates from Ethiopia, DR Congo and Rwanda

**Figure 2:**
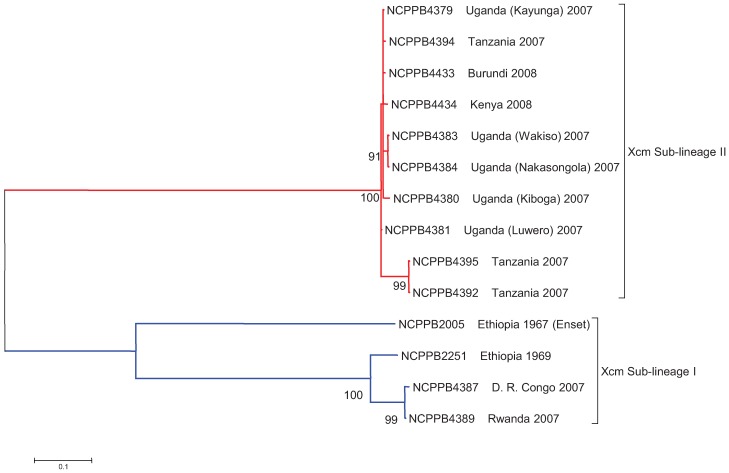
Isolates of *Xcm* from Burundi, Kenya, Tanzania and Uganda comprise a separate sub-lineage that is distinct from isolates from and D. R. Congo, Ethiopia and Rwanda. A maximum likelihood phylogenetic tree was generated as described in the Experimental Section. The position of the root was inferred from the phylogeny presented in Figure 1. The tree is based on 272 SNPs in 14 taxa. Branch lengths are drawn to scale and measured in the number of substitutions per site. Bootstrap values are given as percentages from 500 bootstrap trials.

We next identified SNPs among the *Xcm* and *Xvv* isolates based on BWA [25] alignments of our Illumina sequence data against the *Xcm* NCPPB4381 reference genome sequence (RefSeq: ACHT00000000). Out of 2,908,042 nt over which there was no ambiguity, 2,907,999 were invariant across all isolates; that is the *Xcm* genomes shared at least 99.9985% identity. Based on nucleotides found at 243 polymorphic positions we generated the maximum likelihood phylogenetic tree shown in Figure 2. This clearly delineates the *Xcm* clade into two distinct sub-lineages, I and II. Sub-lineage I include isolates from Ethiopia, DR Congo and Rwanda whilst Sub-lineage II includes isolates from Uganda, Tanzania, Burundi and Kenya. The two sub-lineages are distinguishable from each other by 86 polymorphic positions. These are listed in full in the Supplementary File and in part in Table 3.

**Table 3:** Examples of non-silent single-nucleotide polymorphisms that distinguish *Xcm* sub-lineages I (Ethiopia, DR Congo and Rwanda) and II (Uganda, Kenya, Tanzania, Burundi).

RefSeq Accession	Position	I	II	Locus Tag and Predicted Gene Product
NZ_ACHT01000013	861	g	c	XcampmN_010100000120 putative ISXo8 transposase
NZ_ACHT01000014	6000	a	g	XcampmN_010100000165 putative monovalent cation/H+ antiporter subunit A
NZ_ACHT01000034	8898	t	c	XcampmN_010100000807 putative integrase protein
NZ_ACHT01000045	1261	a	g	XcampmN_010100001162 bifunctional aspartate kinase/diaminopimelate decarboxylase protein
NZ_ACHT01000045	45,548	a	c	XcampmN_010100001377 chemotaxis protein
NZ_ACHT01000059	1907	c	t	XcampmN_010100001687 putative sugar transporter component
NZ_ACHT01000101	995	g	t	XcampmN_010100003517 soluble lytic murein transglycosylase
NZ_ACHT01000104	13,081	t	g	XcampmN_010100003612 GTP-dependent nucleic acid-binding protein EngD
NZ_ACHT01000113	10,410	t	g	XcampmN_010100004062 acetyltransferase (GNAT) family protein
NZ_ACHT01000236	10,652	t	c	XcampmN_010100007340 metallopeptidase
NZ_ACHT01000242	10,465	a	c	XcampmN_010100007585 dihydrolipoamide acetyltransferase
NZ_ACHT01000294	2184	g	t	XcampmN_010100009424 xanthan biosynthesis glucuronosyltransferase GumK
NZ_ACHT01000345	1576	t	c	XcampmN_010100010814 cytochrome C peroxidase
NZ_ACHT01000402	4858	t	c	XcampmN_010100012145 heavy metal transporter
NZ_ACHT01000404	632	g	a	XcampmN_010100012200 tryptophan halogenase
NZ_ACHT01000500	23,584	a	g	XcampmN_010100016057 putative polysaccharide deacetylase
NZ_ACHT01000520	5360	a	g	XcampmN_010100016692 5-methyltetrahydrofolate-homocysteine methyl transferase
NZ_ACHT01000549	7371	a	c	XcampmN_010100018271 two-component system sensor protein
NZ_ACHT01000560	4001	t	c	XcampmN_010100018673 exodeoxyribonuclease III
NZ_ACHT01000590	927	c	t	XcampmN_010100019303 RNA polymerase sigma factor
NZ_ACHT01000626	10,220	t	c	XcampmN_010100019733 putative glutathionylspermidine synthase
NZ_ACHT01000634	2345	t	c	XcampmN_010100019848 beta-mannosidase precursor
NZ_ACHT01000644	2590	g	a	XcampmN_010100020168 two-component system sensor protein
NZ_ACHT01000694	10,665	a	t	XcampmN_010100022153 peptide-acetyl-coenzyme A transporter family protein
NZ_ACHT01000720	19,485	t	c	XcampmN_010100023003 drug:proton antiporter (19121–20371)

**Figure 3:**
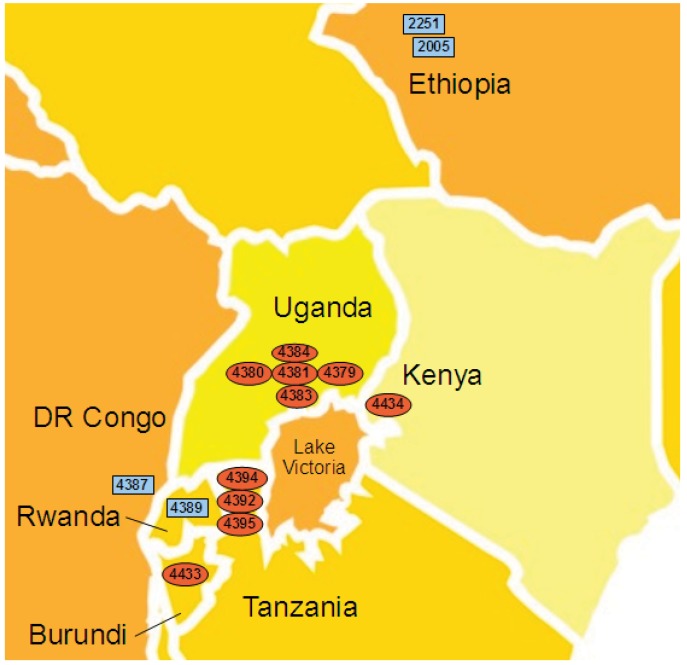
Geographical distribution of the two major sub-lineages of *Xcm*. The approximate geographical locations are indicated for each of the fully sequenced *Xcm* isolates from Ethiopia (NCPPB2005 and NCPPB2251), Uganda (NCPPB4379, NCPPB4380, NCPPB4381, NCPPB4383 and NCPPB4384), Kenya (NCPPB4434), Tanzania (NCPPB4392, NCPPB4394 and NCPPB4395), DR Congo (NCPPB4387), Rwanda (NCPPB4389) and Burundi (NCPPB4433). Blue rectangles indicate locations of isolates belonging to sub-lineage I and red ovals indicate those of sub-lineage II.

The geographic locations of the sequenced *Xcm* isolates are shown in Figure 3. It has been widely assumed that the outbreaks in Uganda, and subsequent outbreaks in neighboring countries, ultimately originated in Ethiopia, with the pathogen perhaps being inadvertently transmitted via international trade. Consistent with this model, the *Xcm* isolates from DR Congo and Rwanda do indeed show extremely high levels of genetic similarity to Ethiopian isolate NCPPB2251. However, the isolates from Uganda, Kenya, Tanzania and Burundi show a distinct genotype, characterized by the 86 consistent SNP differences. This is not consistent with a single introduction from Ethiopia into East Africa.

Our data do not exclude the possibility that the current outbreaks can ultimately be traced back to Ethiopia; it is possible that both lineages I and II are endemic there and it is simply by chance that the two available isolates happen to belong to the DR Congo/Rwanda sub-lineage I. There is an urgent need to collect a range of isolates from Ethiopia and survey their genotypes to ascertain the level of genetic diversity in this pathogen’s presumed centre of origin. Genotyping new isolates should be possible and will be expedited by developing these newly discovered SNPs into PCR based molecular markers. Similarly, there is a pressing need to survey genotypes of a much larger collection of isolates from outbreaks in all the banana growing areas to uncover the routes of geographical spread at a much higher degree of resolution. Ideally a survey of genotypes should be conducted on isolates for which precise details are available on the date and the geographic location at which they are collected.

It should also be noted that although we have categorized the isolates according to the country in which they were collected, paths of transmission may be more influenced by geographical boundaries rather than by political ones. For example, although Rwanda shares two of its borders with Uganda and Tanzania it is somewhat isolated from them by lake and forest.

All of the available isolates from Uganda were collected in 2007 from sites in the central region geographically close to Mukono where the disease was first reported and probably all represent the same single outbreak. It would be interesting to compare these with isolates from outbreaks in Kabale (near Rwanda) or Kasese (near DR Congo). 

All of the available isolates from Tanzania also belonged to sub-lineage II along with those from Uganda. The disease was reported in Tanzania shortly after it was discovered in Uganda and there has been unconfirmed speculation that it may have been inadvertently carried to Tanzania by banana alcohol traders from the Buganda region, close to where the sequenced Ugandan isolates were collected. Our molecular sequence data are consistent with this but do not provide definitive proof.

Although BXW was reported in DR Congo after it was reported in Uganda, the field pictures first sent to Uganda from DR Congo, showed greater devastation. It is not clear where banana *Xanthomonas* wilt occurred first: DR Congo or Uganda. There is a lot of movement of people and bananas from Congo to Rwanda and back, conflicts notwithstanding, and so it is perhaps not surprising that we observe isolates from these two countries belonging to the same sub-lineage I. However, the close relationship between an Ethiopian isolate and those in Rwanda and DRC is not so easily explained unless it is by sampling bias or by one-off international travel; if disease spread was primarily determined by movement of local people and bananas between countries, then we would instead expect isolates from Rwanda, Uganda and DR Congo to cluster together.

### 2.5. Comparison of *Xcm* Isolated from Enset Versus *Xcm* Isolated from Banana

Most of the available isolates of *Xcm* were originally isolated from banana. The exception is NCPPB2005, which was isolated from *Ensete ventricosum* in Ethiopia in 1967. This isolate clearly falls within *Xcm* sub-lineage I (Figure 2). This enset-associated isolate differs from the banana-associated isolates NCPPB2251, NCPPB4387 and NCPPB4389 at 67 SNP positions listed in the Supplementary File. Some examples of these differences are listed in Table 4 and include non-silent polymorphisms in several potential virulence genes (e.g., homologues of *hrpF* and a gene encoding a HopW1 T3SS effector). However, since *Xcm* is able to infect both banana and enset [2,3] it is not clear whether these differences have any biological significance. It would be interesting to survey a much larger sample of isolates from both banana and enset to search for any significant associations between genotype and host species that might reveal adaptation.

**Table 4:** Examples of non-silent single-nucleotide polymorphisms that distinguish NCPPB2251 from banana *versus* NCPPB2005 from enset.

Refseq Accession	Position	NCPPB 2005 (enset)	NCPPB 2251 (Banana)	NCPPB 4389 (Banana)	Locus Tag and Predicted Gene Product
NZ_ACHT01000041	15,615	c	t	t	XcampmN_010100000977 hemolysin III
NZ_ACHT01000072	4507	a	c	c	XcampmN_010100002109 VirB3 protein
NZ_ACHT01000140	1116	c	t	t	XcampmN_010100004536 LacI family transcription regulator
NZ_ACHT01000199	8012	g	t	g	XcampmN_010100006143 type III secreted effector HopW1
NZ_ACHT01000215	3229	c	t	c	XcampmN_010100006660 HrpF protein
NZ_ACHT01000236	9512	c	t	t	XcampmN_010100007340 metallopeptidase
NZ_ACHT01000294	31,553	g	a	a	XcampmN_010100009559 MFS transporter
NZ_ACHT01000303	7530	a	c	c	XcampmN_010100009850 histidine kinase/response regulator hybrid protein
NZ_ACHT01000332	2191	a	g	g	XcampmN_010100010574 putative filamentous hemagglutinin-like protein
NZ_ACHT01000360	1961	a	g	g	XcampmN_010100011266 two-component system sensor protein
NZ_ACHT01000374	12,027	t	c	c	XcampmN_010100011573 Fis family transcriptional regulator
NZ_ACHT01000388	5277	t	g	g	XcampmN_010100011860 AraC family transcriptional regulator
NZ_ACHT01000396	3578	c	g	g	XcampmN_010100011920 catalase
NZ_ACHT01000439	5166	c	g	g	XcampmN_010100013743 ECF subfamily RNA polymerase sigma factor
NZ_ACHT01000532	743	c	t	t	XcampmN_010100017284 beta-glucosidase	
NZ_ACHT01000560	2783	c	t	t	XcampmN_010100018663 molybdopterin biosynthesis	
NZ_ACHT01000668	1036	c	a	a	XcampmN_010100021383 ABC transporter permease	
NZ_ACHT01000690	6284	t	g	g	XcampmN_010100022008 isocitrate dehydrogenase	

### 2.6. Loss of Phage-Associated Genes in Some *Xcm* Isolates

**Figure 4:**
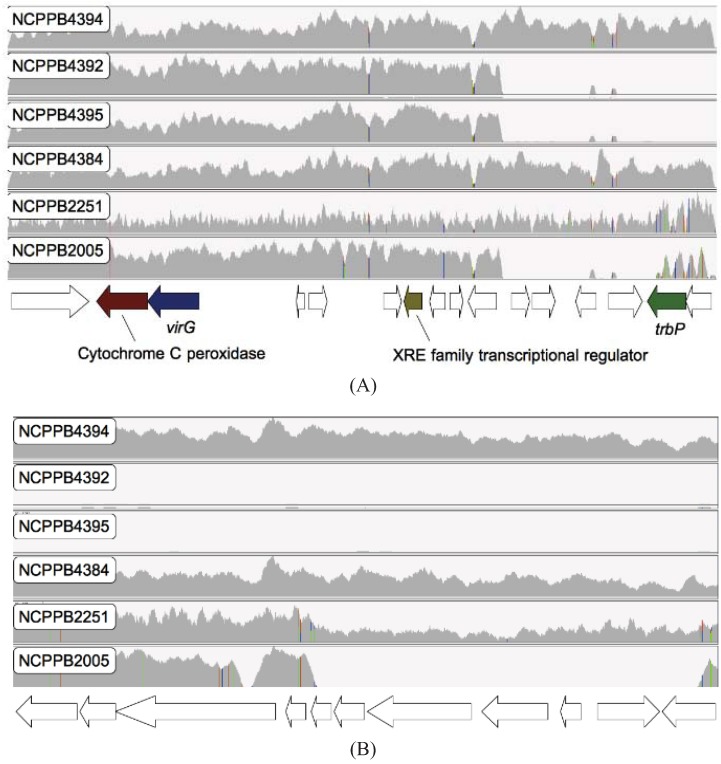
Loss of phage-related genes in two Tanzanian isolates and one Ethiopian isolate of *Xcm*. The figure shows alignments of genomic sequence reads from six *Xcm* isolates *versus* two contigs from the previously published NCPPB4381 genome assembly [12] as viewed in IGV. (**Panel A**): contig_scf_7264_3425_27 (GenBank: ACHT01000345.1); (**Panel B**): contig_scf_7264_3425_29 (GenBank: ACHT01000346.1). Both contigs are contained within genomic scaffold scf_7264_3425 (GenBank: GG699410.1). The vertical axes are the depth of coverage by aligned sequence reads. Coloured vertical bars indicate discrepancies with the NCPPB4381 reference sequence, including SNPs. The horizontal axis is the position on the contig. Positions of predicted genes are indicated below the horizontal axis. Hypothetical genes of unknown function are indicated by white arrows while homologues of characterized genes are indicated by coloured arrows.

In addition to surveying SNPs, we also searched for loss or gain of genes. By aligning sequence reads against the previously published NCPPB4381 genome assembly and systematically comparing gene-coverage in each of the alignments, we were able to identify a genomic region (GenBank: GG699410.1) that showed differential coverage among different isolates of *Xcm* (Figure 4). This region shows significant similarity at the amino acid and nucleotide sequence levels to two previously sequenced phage: *Xanthomonas* phage Cfc1 (RefSeq: NC_001396.1) [26] and *Stenotrophomonas* phage phiSHP2 (GenBank: HM150760.1). Specifically, two Tanzanian isolates (NCPPB4392 and NCPPB4395) appear to have completely lost at least 17 genes from this region, while Ethiopian isolate NCPPB2005 has lost 11 of the same genes. Interestingly, another isolate from the same area of Tanzania (NCPPB4394) appears to have these genes intact as do all the Ugandan, Kenyan, Rwandan and Burundi isolates. Furthermore, there is a high concentration of SNPs in this genomic region. Therefore, it seems likely that this genomic region represents the relic of a phage or similar mobile element that is in the process of degenerating, convergently, in some members of both sub-lineages.

### 2.7. Experimental Validation of Genetic Polymorphisms

One of the major motivations for comparing these genome sequences is to provide a resource for generating molecular markers that can be used epidemiological and biogeographical studies on a much larger panel of isolates without the need for whole-genome sequencing using, for example, the polymerase chain reaction (PCR). Therefore, we used the results of our genome comparisons to design pairs of PCR primers that can be used to distinguish between the two sub-lineages of *Xcm* (Table 5). A similar approach could also be taken to assay other classes of SNPs identified from the genome sequence data.

**Figure 5:**
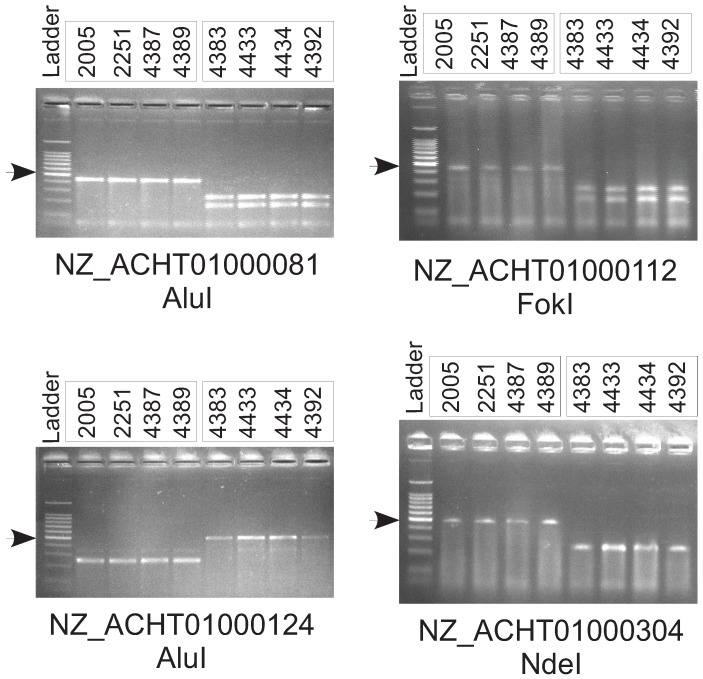
Experimental validation of single-nucleotide polymorphisms (SNPs) between the two sub-lineages of *Xanthomonas campestris* pathovar *musacearum* (*Xcm*). We amplified specific sequence fragments of approximately 500 bp flanking single-nucleotide polymorphisms that we had identified from whole-genome sequencing. Polymerase chain reaction (PCR) was performed on genomic DNA from four isolates from sub-lineage I (NCPPB2005, NCPPB2251, NCPPB4387 and NCPPB4389) and from four isolates of sub-lineage II (NCPPB4383, NCPPB4433, NCPPB4434 and NCPPB4392). The sequences of the PCR primers are given in Table 5. We digested each of the PCR products with a restriction enzyme (AluI, EcoRI, FokI, NdeI or RsaI). We ran the digested PCR products on a 2% agarose gel alongside a 100 bp ladder (Promega G210A) in which the brightest band, marked with a black arrow, indicates 500 bp.

To experimentally validate SNPs, we used an approach based on digestion of PCR products with restriction enzymes. Many of the SNPs that we identified are predicted to fall within restriction sites. For example, position 6150 in RefSeq accession NZ_ACHT0100081 is a G that falls within an AluI restriction site (**AG**↓CT). However, in NCPPB2005 and the other members of sub-lineage I, this G is substituted for an A abolishing the recognition sequence for the AluI restriction enzyme (see Supplementary Files for a figure illustrating this SNP and several others). There are no other AluI sites in the vicinity of this SNP. We generated a pair of primers flanking approximately 250 bp either side of the SNP and amplified this 500 bp sequence by PCR. The two alleles could then be readily distinguished by digestion with AluI (see Figure 5). We used the same approach to design assays for three other SNPs (see Table 5 and Figure 5).

**Table 5:** Polymerase chain reaction (PCR) primers used for distinguishing the two sub-lineages by restriction fragment length polymorphisms.

Primer Sequences	Target Sequence RefSeq Accession Number and Coordinates	Restriction Enzyme
GAGCTCCTGCGCCGATGCGTGAGCGT AAAGGCGGCTATTCTA	NZ_ACHT01000081: 5900–6398	AluI
CGGCGTGGTTTTGCCTTTGCCGTACGG CCTGGCGGTGAT	NZ_ACHT01000112: 10863–11347	FokI
TCACCTGTTCGATGCGGCCGCTACTGG CTGTCGCGGC	NZ_ACHT01000124: 5385–5873	AluI
ATGTTTGCCGATACCTGGATGCGCATG CTTGCCGGTTTCGACGA	NZ_ACHT01000304: 10080–10567	NdeI

## 3. Experimental Section

Bacterial strains were obtained from the National Collection of Plant Pathogenic Bacteria (NCPPB) at FERA. DNA library preparation and genome sequencing using the Illumina GA2x were performed using standard Illumina protocols as previously described [12].

For DNA preparation, bacterial strains were grown overnight at 28 °C in 10 mL King Broth shaken at 200 rpm. Cells were harvested by centrifugation and re-suspended in TE buffer (50 mM Tris-HCl, 40 mM EDTA, pH 8.0). Bacterial cells cultured overnight in Kings Broth were pelleted, lysed with 12 µL of 20 mg/mL lysozyme and RNase at 10 mg/mL and incubated at 25 °C for 10 min. Further lysis was done with 17 µL 10% sodium dodecyl sulfate and incubated on ice for 5min. Proteins were dissolved with 170 µL of 8M ammonium acetate, vortexed vigorously for 30 s centrifuged at 4 °C at maximum speed for 15 min. DNA was precipitated with isopropanol and re-dissolved in 100 μL of 10 mM Tris, pH 8.0, and 1 mM Na2EDTA.

DNA amplification was performed in 30 µL reaction volumes containing 3 µL 10X PCR buffer, 1.2 µL of 50mM MgCl2, 2.4 µL of 2.5 mM dNTP, 1.5 µL of 10 µM each primer, 2ng DNA and 1 U recombinant Taq DNA polymerase. Amplification was performed using a thermocycler with initial denaturation (95 °C, 5 min), followed by 35 cycles of denaturation (95 °C, 0.5 min), annealing (60 °C, 0.5 min) and extension (72 °C, 0.5 min), with a final extension (72 °C, 10 min). The amplified products were electrophoretically separated in 4% (w/v) agarose gel at 80 V for 1 h in TAE buffer and visualized with UV light after staining in ethidium bromide (0.5 µg mL).

Amplified DNA fragments were digested with restriction endonucleases (AluI, Fok or NdeI). The restriction analysis was performed with 2.5 U of the endonuclease using the buffer and temperature recommended by the manufacturers (New England Biolabs). Restriction fragments were separated in a 8% (w/v) agarose gel with 100 bp ladder (Promega, G210A) at 100 V for 1 h in TAE buffer and visualized with UV light after staining in ethidium bromide (0.5 µg mL).

We used BWA [25] to align Illumina sequence reads against a reference genome sequence and used IGV [27] to visualize the alignments (see Figure 4). We used MEGA5 for phylogenetic analyses. *De novo* assembly of Illumina sequence reads was performed using Velvet 1.1.04 [20]. We discarded any sequence reads that contained one or more ‘N’ prior to assembly. 

We used a very conservative approach to infer SNPs from alignments of Illumina reads against the previously published *Xcm* NCPPB4381 reference draft genome assembly. To avoid false positives and false negatives, we only used those regions of the *Xcm* genome with a coverage depth of five or more for every sequenced *Xcm* genome and where there was at least 95% consensus among the sequence reads within each isolate. Just over 60% of the length (2,908,042 out of 4,782,144 nt) of the *Xcm* NCPPB4381 genome fulfilled these two criteria. In other words, for 60% of the *Xcm* genome, there was sufficient quantity and consistency in our data to be almost certain of the sequence in all of the fourteen isolates; for the remaining 40% of the genome, there was some degree of ambiguity in the data for at least one of the isolates. 

Phylogenetic relationships were inferred using a maximum likelihood method based on the Tamura-Nei model [28] conducted using the MEGA5 [29] software package. Bootstrap consensus trees inferred from 500 replicates were taken to represent the evolutionary history of the taxa analyzed. Branches corresponding to partitions reproduced in fewer than 50% bootstrap replicates were collapsed. Initial tree(s) for the heuristic search were obtained automatically as follows. When the number of common sites was <100 or fewer than one quarter of the total number of sites, the maximum parsimony method was used; otherwise the BIONJ method with MCL distance matrix was used. The trees are drawn to scale, with branch lengths measured in the number of substitutions per site. All positions containing gaps and missing data were eliminated. 

## 4. Conclusions

We have deployed high-throughput whole-genome sequencing to explore genetic diversity among isolates of *Xcm*, the bacterial pathogen responsible for BXW, which is devastating banana and plantain crops in East Africa and threatens the food security of millions. To understand the evolution and geographical spread of this newly emerging pathogen, we need molecular markers such as SNPs. Given the high degree of genome sequence identity among isolates (99.9985 %), genome-wide sequencing is the only tractable way of discovering sequence polymorphisms and is beginning to be applied to bacterial phytopathogens [17]. 

The high degree of sequence similarity among isolates indicates a very recent origin of the pathogen. The molecular markers discovered here enabled us to reconstruct the phylogenetic relationships between *Xcm* isolates from diverse geographical locations within the known range of the pathogen in Africa. Interestingly, the isolates fell into two major sub-lineages. This may indicate that there have been at least two separate introductions of *Xcm* into the banana-growing regions around Lake Victoria. This contrasts with the widely held working assumption that *Xcm* spread from Ethiopia to Uganda and thence subsequently into neighboring countries. This view is largely based on the fact that *Xcm* was reported in Ethiopia in the late 1960s, then Uganda in 2001 and only later in other African countries (2004: D. R. Congo, 2005: Rwanda and Tanzania, 2006: Kenya and Burundi). However, it is possible that the disease has existed for some time before being officially reported, especially given the armed conflicts in D. R. Congo and Rwanda at the time. An alternative hypothesis is that all outbreaks in the region can be traced back to a single introduction of inoculum that contained some genetic diversity and that some genetic diversity is maintained within the population. Under that scenario, our results could be explained by stochastic effects of sampling error given such a small number of isolates. Therefore, there is a pressing need to collect and genotype many more isolates, including multiple isolates from within single outbreaks. On the other hand, where we have sequenced multiple isolates from a single geographical area (the five isolates from central Uganda and the three isolates from North Western Tanzania), only a single sub-lineage was observed. This would be an unlikely outcome if both sub-lineages were approximately equally abundant at these sites. Therefore, we currently favour the multiple-introduction model until further isolates and genetic data are available.

## Supplementary Files

Supplementary File 1 PDF-Document (PDF, 376 KB)

Supplementary File 2 PDF-Document (PDF, 198 KB)

Supplementary File 3 PDF-Document (PDF, 132 KB)

Supplementary File 4 PDF-Document (PDF, 357 KB)
