# Supplementary material for: Genome-Wide Sequencing Reveals Two Major Sub-Lineages in the Genetically Monomorphic Pathogen Xanthomonas Campestris Pathovar Musacearum
Source: Genes (Basel). 2012 Jul 4;3(3):361–77. doi: 10.3390/genes3030361 (PMC3902798; doi:10.3390/genes3030361)
Supplement: Supplementary File 1 — PDF-Document (PDF, 376 KB) [file genes-03-00361-s001.pdf]

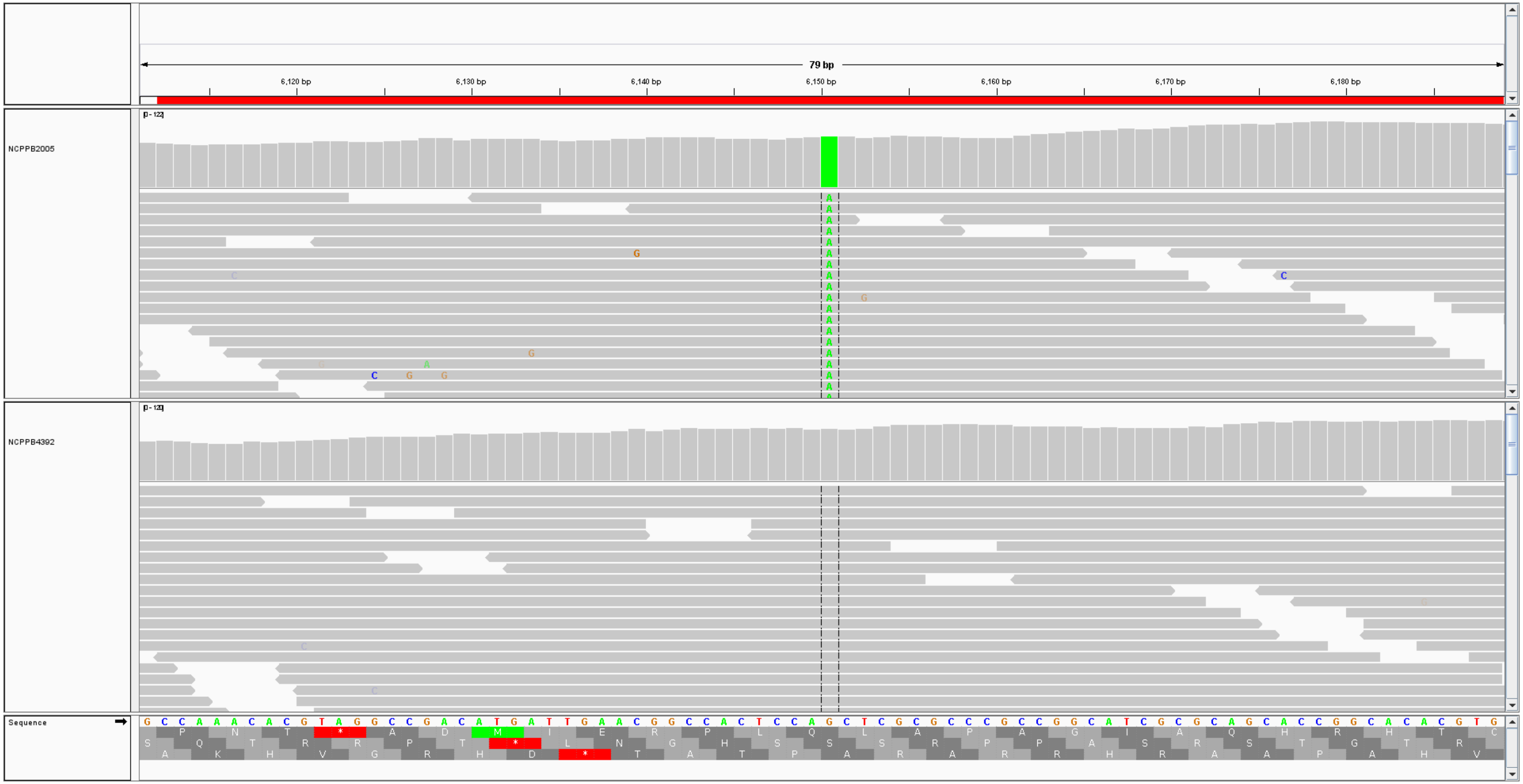

Alignment of genomic sequence reads from NCPPB2005 (sub-lineage I) and NCPPB4392 (sub-lineage II) versus RefSeq:NZ_ACHT0100081. The alignment was generated using BWA and visualised using IGV. The single-nucleotide polymorphism falls within an AluI restriction site (A**G**↓CT) in NCPPB4392 that is abolished in NCPPB2005.

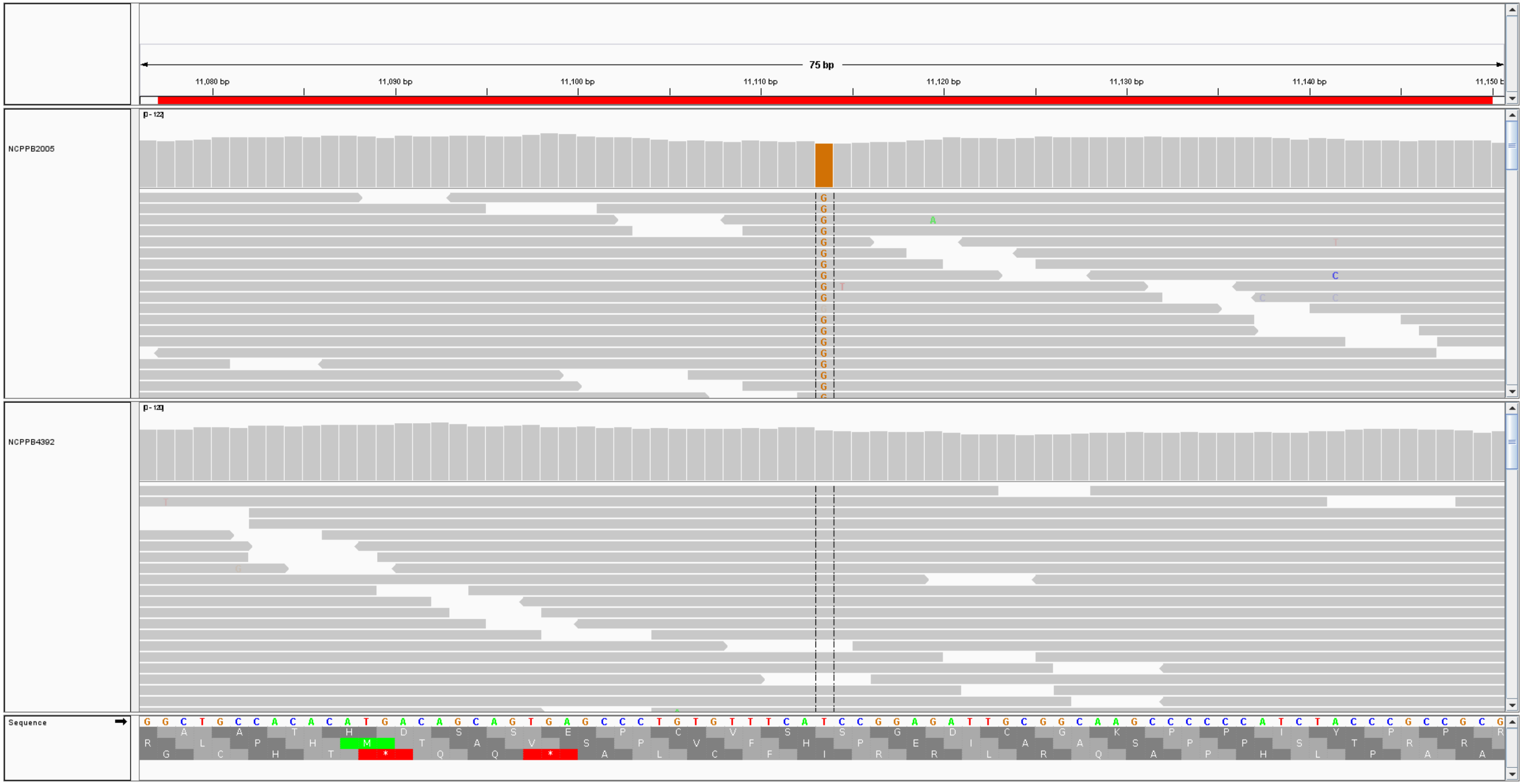

Alignment of genomic sequence reads from NCPPB2005 (sub-lineage I) and NCPPB4392 (sub-lineage II) versus RefSeq:NZ_ACHT0100112. The alignment was generated using BWA and visualised using IGV. The single-nucleotide polymorphism falls within a FokI restriction site ($\downarrow[N]_{13}$CA**T**CC) in NCPPB4392 that is abolished in NCPPB2005.

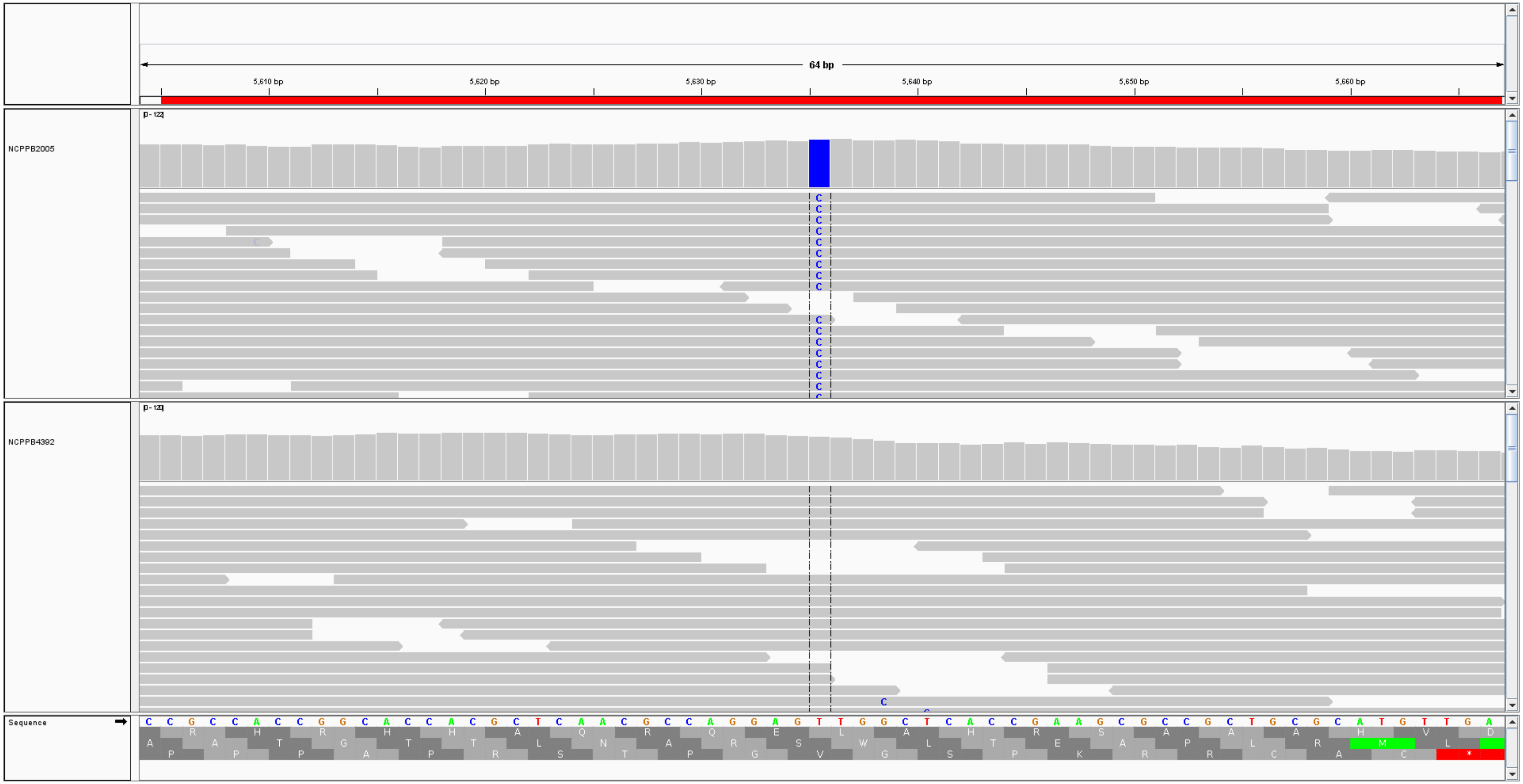

Alignment of genomic sequence reads from NCPPB2005 (sub-lineage I) and NCPPB4392 (sub-lineage II) versus RefSeq:NZ_ACHT0100124. The alignment was generated using BWA and visualised using IGV. The single-nucleotide polymorphism falls generates an AluI restriction site (AG↓**C**T) in NCPP2005 that is absent from NCPPB4392.

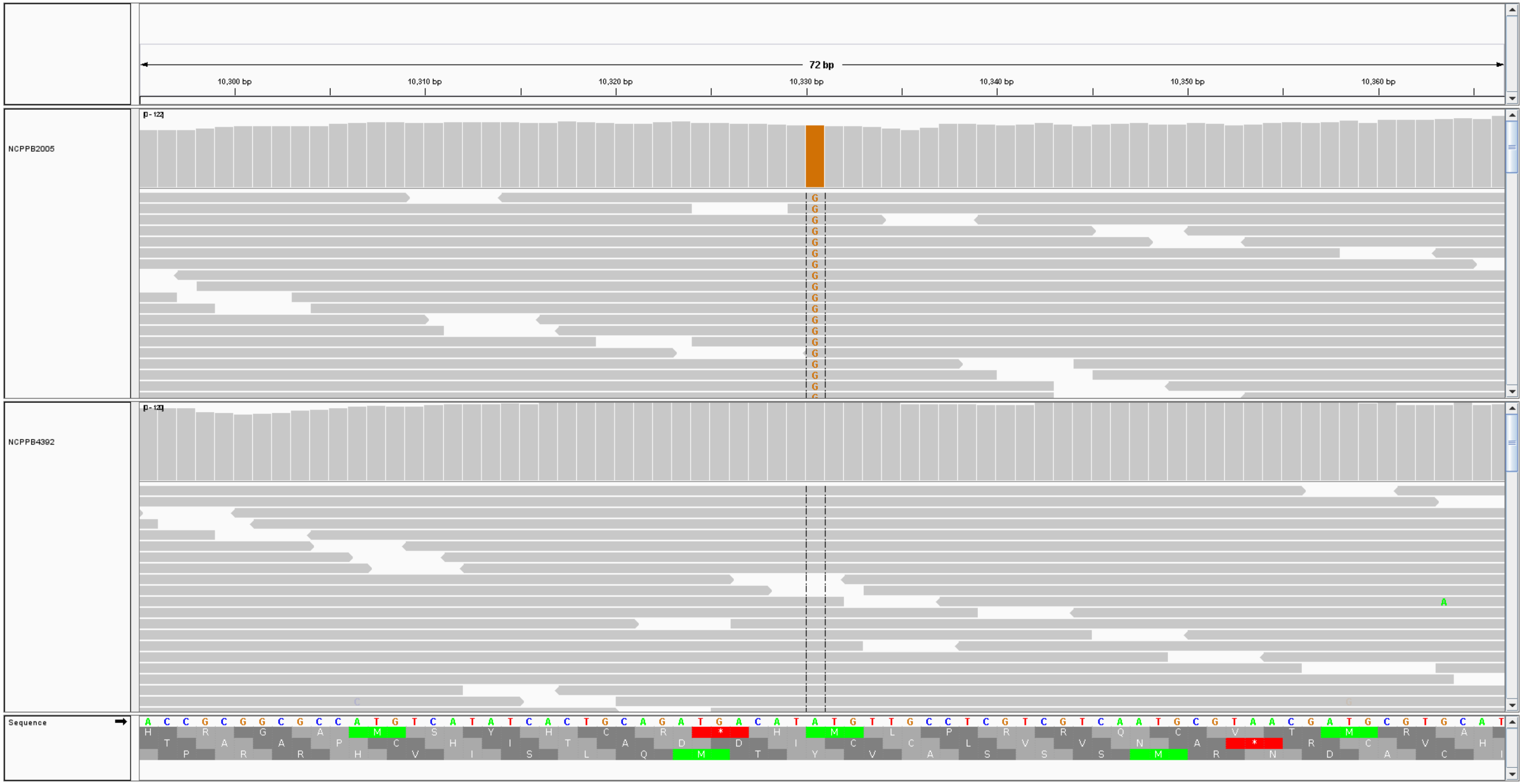

Alignment of genomic sequence reads from NCPPB2005 (sub-lineage I) and NCPPB4392 (sub-lineage II) versus RefSeq:NZ_ACHT0100304. The alignment was generated using BWA and visualised using IGV. The single-nucleotide polymorphism falls within a NdeI restriction site (CA↓TATG) in NCPPB4392 that is abolished in NCPPB2005.
