# Supplementary material for: Genome-Wide Sequencing Reveals Two Major Sub-Lineages in the Genetically Monomorphic Pathogen Xanthomonas Campestris Pathovar Musacearum
Source: Genes (Basel). 2012 Jul 4;3(3):361–77. doi: 10.3390/genes3030361 (PMC3902798; doi:10.3390/genes3030361)
Supplement: Supplementary File 2 — PDF-Document (PDF, 198 KB) [file genes-03-00361-s002.pdf]

Single nucleotide polymorphisms that distinguish Ethiopian Xcm isolates NCPPB2005 (from enset) and NCPPB2251 (from banana)

seq_id	position	2005	2251	4387	4389	4379	4380	4381	4383	4384	4394	4392	4395	4433	4434	genes	silent/non-silent
NZ_ACHT01000713	19319	**G**	**c**	c	c	c	c	c	c	c	c	c	c	c	c	XcampmN_010100022678 putative carbon-nitrogen hyrolase family protein (19176-	non-silent aac -> aaG;
NZ_ACHT01000104	28352	**a**	**G**	a	a	a	a	a	a	a	a	a	a	a	a	XcampmN_010100003657 DNA-directed RNA polymerase subunit beta' (24193-284	non-silent aag -> aGg;
NZ_ACHT01000332	2191	**a**	**G**	G	G	a	a	a	a	a	a	a	a	a	a	XcampmN_010100010574 putative filamentous hemagglutinin-like protein (46-7976	non-silent aca -> Gca;
NZ_ACHT01000666	5161	**C**	**g**	g	g	g	g	g	g	g	g	g	g	g	g	XcampmN_010100021328 hypothetical protein (4518-5378)	non-silent acc -> aGc;
NZ_ACHT01000140	2400	**c**	**A**	c	c	c	c	c	c	c	c	c	c	c	c	XcampmN_010100004541 hypothetical protein (2339-2650)	non-silent act -> aAt;
NZ_ACHT01000468	3962	**T**	**c**	c	c	c	c	c	c	c	c	c	c	c	c	XcampmN_010100014507 relaxation protein (3589-4158)	non-silent aga -> aAa;
NZ_ACHT01000560	2783	**C**	**t**	t	t	t	t	t	t	t	t	t	t	t	t	XcampmN_010100018663 molybdopterin biosynthesis (2151-3404)	non-silent agc -> Ggc;
NZ_ACHT01000668	1036	**C**	**a**	a	a	a	a	a	a	a	a	a	a	a	a	XcampmN_010100021383 ABC transporter permease (1-1466)	non-silent ata -> Cta;
NZ_ACHT01000041	15615	**C**	**t**	t	t	t	t	t	t	t	t	t	t	t	t	XcampmN_010100000977 hemolysin III (15149-15805)	non-silent atc -> aCc;
NZ_ACHT01000538	3594	**t**	**C**	C	C	t	t	t	t	t	t	t	t	t	t	XcampmN_010100017501 hypothetical protein (2378-3724)	non-silent atc -> aCc;
NZ_ACHT01000072	4507	**A**	**c**	c	c	c	c	c	c	c	c	c	c	c	c	XcampmN_010100002109 VirB3 protein (4240-4551)	non-silent atg -> atT;
NZ_ACHT01000694	8404	**T**	**g**	g	g	g	g	g	g	g	g	g	g	g	g	XcampmN_010100022138 orotate phosphoribosyltransferase (7756-8415)	non-silent cac -> caA;
NZ_ACHT01000445	241	**A**	**g**	g	g	g	g	g	g	g	g	g	g	g	g	XcampmN_010100013963 carbonic anhydrase (1-308)	non-silent ccg -> cTg;
NZ_ACHT01000284	672	**A**	**g**	g	g	g	g	g	g	g	g	g	g	g	g	XcampmN_010100009152 hypothetical protein (445-4803)	non-silent ccg -> Tcg;
NZ_ACHT01000101	1035	**T**	**c**	c	c	c	c	c	c	c	c	c	c	c	c	XcampmN_010100003517 soluble lytic murein transglycosylase (1-1051)	non-silent cgc -> cAc;
NZ_ACHT01000551	5522	**c**	**T**	c	c	c	c	c	c	c	c	c	c	c	c	XcampmN_010100018453 excinuclease ABC subunit A (4595-6156)	non-silent cgc -> Tgc;
NZ_ACHT01000205	131	**g**	**A**	A	A	g	g	g	g	g	g	g	g	g	g	XcampmN_010100006318 putative integrase (1-859)	non-silent cgt -> cAt;
NZ_ACHT01000296	5624	**a**	**C**	C	C	a	a	a	a	a	a	a	a	a	a	XcampmN_010100009626 hypothetical protein (5581-6774)	non-silent ctg -> cGg;
NZ_ACHT01000256	13371	**g**	**C**	C	C	g	g	g	g	g	g	g	g	g	g	XcampmN_010100008595 hypothetical protein (12964-13470)	non-silent ctg -> Gtg;
NZ_ACHT01000396	3578	**c**	**G**	G	G	c	c	c	c	c	c	c	c	c	c	XcampmN_010100011920 catalase (3375-5483)	non-silent gaa -> Caa;
NZ_ACHT01000236	9512	**C**	**t**	t	t	t	t	t	t	t	t	t	t	t	t	XcampmN_010100007340 metallopeptidase (8644-10743)	non-silent gaa -> gGa;
NZ_ACHT01000468	3975	**T**	**c**	c	c	c	c	c	c	c	c	c	c	c	c	XcampmN_010100014507 relaxation protein (3589-4158)	non-silent gac -> Aac;
NZ_ACHT01000080	976	**c**	**T**	T	T	c	c	c	c	c	c	c	c	c	c	XcampmN_010100002427 histidine kinase/response regulator hybrid protein (1-218	non-silent gac -> Aac;
NZ_ACHT01000478	1287	**c**	**T**	T	T	c	c	c	c	c	c	c	c	c	c	XcampmN_010100014662 phage-related tail protein (1111-1320)	non-silent gac -> Aac;
NZ_ACHT01000303	7530	**A**	**c**	c	c	c	c	c	c	c	c	c	c	c	c	XcampmN_010100009850 histidine kinase/response regulator hybrid protein (7183-	non-silent gac -> gaA;
NZ_ACHT01000648	23525	**a**	**T**	T	T	a	a	a	a	a	a	a	a	a	a	XcampmN_010100020488 DNA polymerase I (21271-24072)	non-silent gac -> gTc;
NZ_ACHT01000690	6284	**T**	**g**	g	g	g	g	g	g	g	g	g	g	g	g	XcampmN_010100022008 isocitrate dehydrogenase%2C NADP-dependent (5354-	non-silent gac -> Tac;
NZ_ACHT01000439	5166	**C**	**g**	g	g	g	g	g	g	g	g	g	g	g	g	XcampmN_010100013743 ECF subfamily RNA polymerase sigma factor (5037-556	non-silent gag -> Cag;
NZ_ACHT01000044	2934	**T**	**c**	c	c	c	c	c	c	c	c	c	c	c	c	XcampmN_010100001152 hypothetical protein (2671-3549)	non-silent gat -> Aat;
NZ_ACHT01000468	3862	**a**	**T**	T	T	a	a	a	a	a	a	a	a	a	a	XcampmN_010100014507 relaxation protein (3589-4158)	non-silent gat -> gaA;
NZ_ACHT01000256	3474	**A**	**g**	g	g	g	g	g	g	g	g	g	g	g	g	XcampmN_010100008560 hypothetical protein (2322-5207)	non-silent gca -> Aca;
NZ_ACHT01000382	10916	**g**	**T**	T	T	g	g	g	g	g	g	g	g	g	g	XcampmN_010100011693 2-methylisocitrate lyase (10912-11808)	non-silent gca -> gAa;
NZ_ACHT01000361	4094	**A**	**c**	c	c	c	c	c	c	c	c	c	c	c	c	XcampmN_010100011278 transcription-related protein (3645-6008)	non-silent gca -> Tca;
NZ_ACHT01000405	6174	**A**	**g**	g	g	g	g	g	g	g	g	g	g	g	g	XcampmN_010100012295 ATP-dependent DNA helicase DinG (4482-6617)	non-silent gcc -> Acc;
NZ_ACHT01000242	12462	**g**	**A**	A	A	g	g	g	g	g	g	g	g	g	g	XcampmN_010100007595 dihydrolipoamide dehydrogenase (11334-13157)	non-silent gcc -> Acc;
NZ_ACHT01000140	1116	**c**	**T**	T	T	c	c	c	c	c	c	c	c	c	c	XcampmN_010100004536 LacI family transcription regulator (1016-2011)	non-silent gcc -> gTc;
NZ_ACHT01000532	743	**c**	**T**	T	T	c	c	c	c	c	c	c	c	c	c	XcampmN_010100017284 beta-glucosidase (1-1772)	non-silent gcc -> gTc;
NZ_ACHT01000725	3818	**c**	**A**	A	A	c	c	c	c	c	c	c	c	c	c	XcampmN_010100023135 Putative signal protein with GGDEF and EAL domains (1	non-silent gcc -> Tcc;
NZ_ACHT01000294	31553	**g**	**A**	A	A	g	g	g	g	g	g	g	g	g	g	XcampmN_010100009559 MFS transporter (30245-31570)	non-silent gcg -> Acg;
NZ_ACHT01000101	2893	**T**	**c**	c	c	c	c	c	c	c	c	c	c	c	c	XcampmN_010100003522 TonB-dependent receptor (1308-4187)	non-silent gcg -> gTg;
NZ_ACHT01000044	3736	**A**	**g**	g	g	g	g	g	g	g	g	g	g	g	g	XcampmN_010100001157 bifunctional aspartate kinase/diaminopimelate decarboxy	non-silent gct -> Act;
NZ_ACHT01000374	12027	**T**	**c**	c	c	c	c	c	c	c	c	c	c	c	c	XcampmN_010100011573 Fis family transcriptional regulator (11777-12145)	non-silent gct -> gTt;
NZ_ACHT01000033	777	**c**	**A**	A	A	c	c	c	c	c	c	c	c	c	c	XcampmN_010100000737 hypothetical protein (463-1539)	non-silent gct -> Tct;
NZ_ACHT01000388	5277	**T**	**g**	g	g	g	g	g	g	g	g	g	g	g	g	XcampmN_010100011860 AraC family transcriptional regulator (4946-5671)	non-silent ggc -> gTc;
NZ_ACHT01000642	197	**A**	**c**	c	c	c	c	c	c	c	c	c	c	c	c	XcampmN_010100020088 hypothetical protein (37-675)	non-silent ggc -> gTc;
NZ_ACHT01000250	10340	**g**	**C**	C	C	g	g	g	g	g	g	g	g	g	g	XcampmN_010100008245 hypothetical protein (9986-10390)	non-silent ggg -> Cgg;
NZ_ACHT01000215	3229	**c**	**T**	c	c	c	c	c	c	c	c	c	c	c	c	XcampmN_010100006660 HrpF protein (2601-5183)	non-silent ggg -> gAg;
NZ_ACHT01000493	3280	**A**	**g**	g	g	g	g	g	g	g	g	g	g	g	g	XcampmN_010100015452 hypothetical protein (2929-3501)	non-silent ggt -> Agt;
NZ_ACHT01000661	4425	**c**	**T**	T	T	c	c	c	c	c	c	c	c	c	c	XcampmN_010100020913 branched-chain amino acid aminotransferase (4229-531	non-silent ggt -> gAt;
NZ_ACHT01000028	7775	**A**	**c**	c	c	c	c	c	c	c	c	c	c	c	c	XcampmN_010100000587 ribosomal protein alanine acetyltransferase (7614-8207)	non-silent tac -> taA;
NZ_ACHT01000120	419	**A**	**c**	c	c	c	c	c	c	c	c	c	c	c	c	XcampmN_010100004174 acyl carrier protein phosphodiesterase (162-746)	non-silent tac -> taA;
NZ_ACHT01000482	10486	**c**	**T**	T	T	c	c	c	c	c	c	c	c	c	c	XcampmN_010100015002 hypothetical protein (10002-10820)	non-silent tcg -> tTg;
NZ_ACHT01000346	5977	**A**	**c**	c	c	c	c	c	c	c	c	A	A	c	c	XcampmN_010100010944 hypothetical protein (5604-6218)	non-silent tct -> tAt;
NZ_ACHT01000360	1961	**A**	**g**	g	g	g	g	g	g	g	g	g	g	g	g	XcampmN_010100011266 two-component system sensor protein (1630-2210)	non-silent tgg -> tAg;
NZ_ACHT01000199	8012	**g**	**T**	g	g	g	g	g	g	g	g	g	g	g	g	XcampmN_010100006143 type III secreted effector hopPmaA (6887-8158)	non-silent ttc -> ttA;
NZ_ACHT01000104	11913	**t**	**G**	t	t	t	t	t	t	t	t	t	t	t	t	XcampmN_010100003602 50S ribosomal protein L25/general stress protein Ctc (11	non-silent ttg -> tGg;

Single nucleotide polymorphisms that distinguish Ethiopian Xcm isolates NCPPB2005 (from enset) and NCPPB2251 (from banana)

seq_id	position	2005	2251	4387	4389	4379	4380	4381	4383	4384	4394	4392	4395	4433	4434	genes	silent/non-silent
NZ_ACHT01000175	2617	T	c	c	c	c	c	c	c	c	c	c	c	c	c	XcampmN_010100005213 hypothetical protein (1862-2704)	silent aac -> aaT;
NZ_ACHT01000508	35194	T	c	c	c	c	c	c	c	c	c	c	c	c	c	XcampmN_010100016342 disulphide-isomerase (34751-36331)	silent aac -> aaT;
NZ_ACHT01000186	11109	C	g	g	g	g	g	g	g	g	g	g	g	g	g	XcampmN_010100005558 orotidine 5'-phosphate decarboxylase (10740-11471)	silent acc -> acG;
NZ_ACHT01000382	21607	A	g	g	g	g	g	g	g	g	g	g	g	g	g	XcampmN_010100011748 3-ketoacyl-(acyl-carrier-protein) reductase (21142-21885	silent acc -> acT;
NZ_ACHT01000480	1808	C	g	g	g	g	g	g	g	g	g	g	g	g	g	XcampmN_010100014847 hypothetical protein (1335-1976)	silent acg -> acC;
NZ_ACHT01000043	10914	c	T	T	T	c	c	c	c	c	c	c	c	c	c	XcampmN_010100001107 putative siderophore biosynthesis protein (9991-11760)	silent agc -> agT;
NZ_ACHT01000089	7071	g	A	A	A	g	g	g	g	g	g	g	g	g	g	XcampmN_010100002867 GGDEF family protein (7068-7727)	silent agc -> agT;
NZ_ACHT01000510	2480	g	A	A	A	g	g	g	g	g	g	g	g	g	g	XcampmN_010100016452 drug:H+ antiporter-1 family protein (2006-3271)	silent agc -> agT;
NZ_ACHT01000089	30693	A	c	c	c	c	c	c	c	c	c	c	c	c	c	XcampmN_010100002947 hypothetical protein (30250-31116)	silent atc -> atA;
NZ_ACHT01000518	4688	g	A	A	A	g	g	g	g	g	g	g	g	g	g	XcampmN_010100016582 sulfate transporter (3671-5140)	silent atc -> atT;
NZ_ACHT01000268	5921	T	c	c	c	c	c	c	c	c	c	c	c	c	c	XcampmN_010100008867 general secretory pathway related protein (5108-6922)	silent cag -> caA;
NZ_ACHT01000045	46634	c	T	T	T	c	c	c	c	c	c	c	c	c	c	XcampmN_010100001382 chemotaxis protein (45818-47971)	silent cag -> caA;
NZ_ACHT01000520	15004	c	A	A	A	c	c	c	c	c	c	c	c	c	c	XcampmN_010100016722 hypothetical protein (14338-15102)	silent ccg -> ccT;
NZ_ACHT01000045	12533	c	A	A	A	c	c	c	c	c	c	c	c	c	c	XcampmN_010100001212 putative xanthine dehydrogenase iron-sulfur-binding sub	silent cgc -> cgA;
NZ_ACHT01000459	3831	T	c	c	c	c	c	c	c	c	c	c	c	c	c	XcampmN_010100014168 aspartyl-tRNA synthetase (2080-3846)	silent cgc -> cgT;
NZ_ACHT01000438	15721	A	g	g	g	g	g	g	g	g	g	g	g	g	g	XcampmN_010100013693 transposase IS3/IS911 family protein (15704-15877)	silent cta -> Tta;
NZ_ACHT01000223	8945	c	T	c	c	c	c	c	c	c	c	c	c	c	c	XcampmN_010100006945 RNA polymerase ECF-type sigma factor (8585-9022)	silent ctg -> ctA;
NZ_ACHT01000420	5559	c	G	G	G	c	c	c	c	c	c	c	c	c	c	XcampmN_010100013024 putative 2OG-Fe(II) oxygenase superfamily protein (479	silent ctg -> ctC;
NZ_ACHT01000479	19146	A	c	c	c	c	c	c	c	c	c	c	c	c	c	XcampmN_010100014832 putative glycosyltransferase (18078-19262)	silent ctg -> ctT;
NZ_ACHT01000123	3457	T	c	c	c	c	c	c	c	c	c	c	c	c	c	XcampmN_010100004234 hypothetical protein (3202-3477)	silent ctg -> Ttg;
NZ_ACHT01000480	7300	T	c	c	c	c	c	c	c	c	c	c	c	c	c	XcampmN_010100014877 hypothetical protein (7267-7509)	silent ctg -> Ttg;
NZ_ACHT01000539	13258	A	g	g	g	g	g	g	g	g	g	g	g	g	g	XcampmN_010100017736 2'-5' RNA ligase (13049-13651)	silent ctg -> Ttg;
NZ_ACHT01000541	14901	T	c	c	c	c	c	c	c	c	c	c	c	c	c	XcampmN_010100017896 putative sensor protein (14658-15320)	silent ctg -> Ttg;
NZ_ACHT01000550	11623	T	c	c	c	c	c	c	c	c	c	c	c	c	c	XcampmN_010100018361 HrpG (11458-12249)	silent ctg -> Ttg;
NZ_ACHT01000721	1937	c	T	c	c	c	c	c	c	c	c	c	c	c	c	XcampmN_010100023025 dihydroorotase (896-2245)	silent ctg -> Ttg;
NZ_ACHT01000099	23369	c	T	T	T	c	c	c	c	c	c	c	c	c	c	XcampmN_010100003437 N-acetylglucosaminidase (22499-24895)	silent ctg -> Ttg;
NZ_ACHT01000378	1614	T	c	c	c	c	c	c	c	c	c	c	c	c	c	XcampmN_010100011643 conjugal transfer relaxosome component TraJ (1608-19	silent gag -> gaA;
NZ_ACHT01000666	4108	c	T	c	c	c	c	c	c	c	c	c	c	c	c	XcampmN_010100021323 hypothetical protein (4027-4458)	silent gag -> gaA;
NZ_ACHT01000229	981	g	A	A	A	g	g	g	g	g	g	g	g	g	g	XcampmN_010100007125 leucyl-tRNA synthetase (76-2718)	silent gag -> gaA;
NZ_ACHT01000564	18074	c	T	T	T	c	c	c	c	c	c	c	c	c	c	XcampmN_010100018848 ATP-dependent Clp protease subunit (16745-19027)	silent gag -> gaA;
NZ_ACHT01000468	3715	g	C	C	C	g	g	g	g	g	g	g	g	g	g	XcampmN_010100014507 relaxation protein (3589-4158)	silent gcc -> gcG;
NZ_ACHT01000515	1945	A	g	g	g	g	g	g	g	g	g	g	g	g	g	XcampmN_010100016502 23S rRNA m(2)G2445 methyltransferase (1322-2739)	silent gcc -> gcT;
NZ_ACHT01000215	9600	T	c	c	c	c	c	c	c	c	c	c	c	c	c	XcampmN_010100006680 HpaB protein (9387-9857)	silent gcg -> gcA;
NZ_ACHT01000733	5016	T	c	c	c	c	c	c	c	c	c	c	c	c	c	XcampmN_010100023330 hypothetical protein (4768-6317)	silent gcg -> gcA;
NZ_ACHT01000713	36931	A	c	c	c	c	c	c	c	c	c	c	c	c	c	XcampmN_010100022743 segregation and condensation protein A (36554-37459)	silent ggc -> ggA;
NZ_ACHT01000468	3898	A	g	g	g	g	g	g	g	g	g	g	g	g	g	XcampmN_010100014507 relaxation protein (3589-4158)	silent ggc -> ggT;
NZ_ACHT01000245	41520	A	c	c	c	c	c	c	c	c	c	c	c	c	c	XcampmN_010100008015 hypothetical protein (40362-43199)	silent ggg -> ggT;
NZ_ACHT01000522	830	c	A	A	A	c	c	c	c	c	c	c	c	c	c	XcampmN_010100016839 2-oxoglutarate dehydrogenase E1 component (738-1080	silent gtc -> gtA;
NZ_ACHT01000468	3955	T	c	c	c	c	c	c	c	c	c	c	c	c	c	XcampmN_010100014507 relaxation protein (3589-4158)	silent gtg -> gtA;
NZ_ACHT01000495	13033	A	g	g	g	g	g	g	g	g	g	g	g	g	g	XcampmN_010100015547 superoxide dismutase (12641-13138)	silent gtg -> gtA;
NZ_ACHT01000675	9320	c	T	T	T	c	c	c	c	c	c	c	c	c	c	XcampmN_010100021653 cell division protein FtsA (8864-10099)	silent gtg -> gtA;
NZ_ACHT01000468	3931	A	c	c	c	c	c	c	c	c	c	c	c	c	c	XcampmN_010100014507 relaxation protein (3589-4158)	silent gtg -> gtT;
NZ_ACHT01000209	297	T	c	c	c	c	c	c	c	c	c	c	c	c	c	XcampmN_010100006463 protocatechuate 3%2C4-dioxygenase beta chain (1-684)	silent tac -> taT;
NZ_ACHT01000442	14471	C	t	t	t	t	t	t	t	t	t	t	t	t	t	XcampmN_010100013898 hypothetical protein (14031-14639)	silent tat -> taC;
NZ_ACHT01000091	253	c	T	T	T	c	c	c	c	c	c	c	c	c	c	XcampmN_010100003067 putative secreted protein (20-4942)	silent ttc -> ttT;
NZ_ACHT01000436	3952	a	G	G	G	a	a	a	a	a	a	a	a	a	a	XcampmN_010100013633 XopX effector protein (2588-4771)	silent ttg -> Ctg;
NZ_ACHT01000023	16591	G	t	t	t	t	t	t	t	t	t	t	t	t	t	Intergenic	Intergenic
NZ_ACHT01000060	1984	C	a	a	a	a	a	a	a	a	a	a	a	a	a	Intergenic	Intergenic
NZ_ACHT01000064	1939	C	t	t	t	t	t	t	t	t	t	t	t	t	t	Intergenic	Intergenic
NZ_ACHT01000178	5582	T	g	g	g	g	g	g	g	g	g	g	g	g	g	Intergenic	Intergenic
NZ_ACHT01000196	1762	A	c	c	c	c	c	c	c	c	c	c	c	c	c	Intergenic	Intergenic
NZ_ACHT01000450	1683	G	a	a	a	a	a	a	a	a	a	a	a	a	a	Intergenic	Intergenic
NZ_ACHT01000491	4107	T	g	g	g	g	g	g	g	g	g	g	g	g	g	Intergenic	Intergenic
NZ_ACHT01000491	12761	T	c	c	c	c	c	c	c	c	c	c	c	c	c	Intergenic	Intergenic

Single nucleotide polymorphisms that distinguish Ethiopian Xcm isolates NCPPB2005 (from enset) and NCPPB2251 (from banana)

seq_id	position	2005	2251	4387	4389	4379	4380	4381	4383	4384	4394	4392	4395	4433	4434	genes	silent/non-silent
NZ_ACHT01000520	3959	**T**	**c**	c	c	c	c	c	c	c	c	c	c	c	c	Intergenic	Intergenic
NZ_ACHT01000666	5784	**C**	**g**	g	g	g	g	g	g	g	g	g	g	g	g	Intergenic	Intergenic
NZ_ACHT01000683	20156	**T**	**g**	g	g	g	g	g	g	g	g	g	g	g	g	Intergenic	Intergenic
NZ_ACHT01000089	17864	**g**	**A**	g	g	g	g	g	g	g	g	g	g	g	g	Intergenic	Intergenic
NZ_ACHT01000499	17684	**c**	**T**	c	c	c	c	c	c	c	c	c	c	c	c	Intergenic	Intergenic
NZ_ACHT01000609	10047	**g**	**A**	g	g	g	g	g	g	g	g	g	g	g	g	Intergenic	Intergenic
NZ_ACHT01000726	10603	**a**	**G**	a	a	a	a	a	a	a	a	a	a	a	a	Intergenic	Intergenic
NZ_ACHT01000060	2002	**g**	**C**	C	C	g	g	g	g	g	g	g	g	g	g	Intergenic	Intergenic
NZ_ACHT01000091	8390	**c**	**A**	A	A	c	c	c	c	c	c	c	c	c	c	Intergenic	Intergenic
NZ_ACHT01000138	21001	**c**	**T**	T	T	c	c	c	c	c	c	c	c	c	c	Intergenic	Intergenic
NZ_ACHT01000413	4760	**g**	**A**	A	A	g	g	g	g	g	g	g	g	g	g	Intergenic	Intergenic
NZ_ACHT01000418	297	**t**	**G**	G	G	t	t	t	t	t	t	t	t	t	t	Intergenic	Intergenic
NZ_ACHT01000421	8792	**g**	**T**	T	T	g	g	g	g	g	g	g	g	g	g	Intergenic	Intergenic
NZ_ACHT01000478	8851	**g**	**A**	A	A	g	g	g	g	g	g	g	g	g	g	Intergenic	Intergenic
NZ_ACHT01000508	22731	**g**	**A**	A	A	g	g	g	g	g	g	g	g	g	g	Intergenic	Intergenic
NZ_ACHT01000538	20186	**t**	**C**	C	C	t	t	t	t	t	t	t	t	t	t	Intergenic	Intergenic
NZ_ACHT01000549	1367	**g**	**A**	A	A	g	g	g	g	g	g	g	g	g	g	Intergenic	Intergenic
NZ_ACHT01000580	604	**g**	**A**	A	A	g	g	g	g	g	g	g	g	g	g	Intergenic	Intergenic
NZ_ACHT01000640	6733	**a**	**G**	G	G	a	a	a	a	a	a	a	a	a	a	Intergenic	Intergenic
NZ_ACHT01000719	10514	**c**	**T**	T	T	c	c	c	c	c	c	c	c	c	c	Intergenic	Intergenic
