# Supplementary material for: Genome-Wide Sequencing Reveals Two Major Sub-Lineages in the Genetically Monomorphic Pathogen Xanthomonas Campestris Pathovar Musacearum
Source: Genes (Basel). 2012 Jul 4;3(3):361–77. doi: 10.3390/genes3030361 (PMC3902798; doi:10.3390/genes3030361)
Supplement: Supplementary File 3 — PDF-Document (PDF, 132 KB) [file genes-03-00361-s003.pdf]

Single-nucleotide polymorphisms that distinguish the two sub-lineages of Xcm

RefSeq accession	position	2005	2251	4387	4389	4379	4380	4381	4383	4384	4394	4392	4395	4433	4434	genes	silent/non-silent
NZ_ACHT01000045	19682	G	G	G	G	a	a	a	a	a	a	a	a	a	a	XcampmN_010100001247 hypothetical protein (18823-20073)	non-silent aac -> aGc;
NZ_ACHT01000284	4251	C	C	C	C	t	t	t	t	t	t	t	t	t	t	XcampmN_010100009152 hypothetical protein (445-4803)	non-silent aca -> Gca;
NZ_ACHT01000242	10465	A	A	A	A	c	c	c	c	c	c	c	c	c	c	XcampmN_010100007585 dihydrolipoamide acetyltransferase (8913-10673)	non-silent acg -> aAg;
NZ_ACHT01000644	2590	G	G	G	G	a	a	a	a	a	a	a	a	a	a	XcampmN_010100020168 two-component system sensor protein (1-2672)	non-silent act -> Gct;
NZ_ACHT01000101	995	G	G	G	G	t	t	t	t	t	t	t	t	t	t	XcampmN_010100003517 soluble lytic murein transglycosylase (1-1051)	non-silent aga -> agC;
NZ_ACHT01000045	45548	A	A	A	A	c	c	c	c	c	c	c	c	c	c	XcampmN_010100001377 chemotaxis protein (45292-45807)	non-silent agc -> aTc;
NZ_ACHT01000296	6204	C	C	C	C	t	t	t	t	t	t	t	t	t	t	XcampmN_010100009626 hypothetical protein (5581-6774)	non-silent atg -> Gtg;
NZ_ACHT01000013	861	G	G	G	G	c	c	c	c	c	c	c	c	c	c	XcampmN_010100000120 putative ISXo8 transposase (24-929)	non-silent cat -> Gat;
NZ_ACHT01000626	10220	T	T	T	T	c	c	c	c	c	c	c	c	c	c	XcampmN_010100019733 putative glutathionylspermidine synthase (9136-10302)	non-silent cct -> cTt;
NZ_ACHT01000549	7371	A	A	A	A	c	c	c	c	c	c	c	c	c	c	XcampmN_010100018271 two-component system sensor protein (6453-9626)	non-silent cgc -> Agc;
NZ_ACHT01000014	6000	A	A	A	A	g	g	g	g	g	g	g	g	g	g	XcampmN_010100000165 putative monovalent cation/H+ antiporter subunit A (4687-7515)	non-silent cgg -> Tgg;
NZ_ACHT01000388	4711	T	T	T	T	g	g	g	g	g	g	g	g	g	g	XcampmN_010100011855 hypothetical protein (4350-4814)	non-silent cgt -> cTt;
NZ_ACHT01000590	927	C	C	C	C	t	t	t	t	t	t	t	t	t	t	XcampmN_010100019303 RNA polymerase sigma factor (518-1285)	non-silent ctc -> cCc;
NZ_ACHT01000407	2050	G	G	G	G	t	t	t	t	t	t	t	t	t	t	XcampmN_010100012345 hypothetical protein (1446-3047)	non-silent ctc -> cGc;
NZ_ACHT01000175	16953	G	G	G	G	c	c	c	c	c	c	c	c	c	c	XcampmN_010100005303 hypothetical protein (16770-16985)	non-silent ctc -> Gtc;
NZ_ACHT01000689	3576	A	A	A	A	g	g	g	g	g	g	g	g	g	g	XcampmN_010100021983 crispr-associated protein%2C Csd1 family (3517-5361)	non-silent ctc -> Ttc;
NZ_ACHT01000404	632	G	G	G	G	a	a	a	a	a	a	a	a	a	a	XcampmN_010100012200 tryptophan halogenase (622-2175)	non-silent ctt -> cCt;
NZ_ACHT01000474	4733	G	G	G	G	a	a	a	a	a	a	a	a	a	a	XcampmN_010100014587 putative secreted protein (3265-5007)	non-silent gac -> gGc;
NZ_ACHT01000402	4858	T	T	T	T	c	c	c	c	c	c	c	c	c	c	XcampmN_010100012145 heavy metal transporter (4406-5386)	non-silent gat -> Aat;
NZ_ACHT01000034	8898	T	T	T	T	c	c	c	c	c	c	c	c	c	c	XcampmN_010100000807 putative integrase protein (8798-8935)	non-silent gca -> gTa;
NZ_ACHT01000113	10410	T	T	T	T	g	g	g	g	g	g	g	g	g	g	XcampmN_010100004062 acetyltransferase (GNAT) family protein (10166-10723)	non-silent gcc -> gAc;
NZ_ACHT01000267	349	T	T	T	T	c	c	c	c	c	c	c	c	c	c	XcampmN_010100008857 hypothetical protein (9-1705)	non-silent gcc -> gTc;
NZ_ACHT01000500	23584	A	A	A	A	g	g	g	g	g	g	g	g	g	g	XcampmN_010100016057 putative polysaccharide deacetylase (23526-24416)	non-silent gcc -> gTc;
NZ_ACHT01000634	2345	T	T	T	T	c	c	c	c	c	c	c	c	c	c	XcampmN_010100019848 beta-mannosidase precursor (2314-3761)	non-silent gcc -> gTc;
NZ_ACHT01000045	1261	A	A	A	A	g	g	g	g	g	g	g	g	g	g	XcampmN_010100001162 bifunctional aspartate kinase/diaminopimelate decarboxylase protein (	non-silent gcg -> Acg;
NZ_ACHT01000560	4001	T	T	T	T	c	c	c	c	c	c	c	c	c	c	XcampmN_010100018673 exodeoxyribonuclease III (3924-4913)	non-silent gct -> Act;
NZ_ACHT01000268	14452	T	T	T	T	g	g	g	g	g	g	g	g	g	g	XcampmN_010100008887 hypothetical protein (12246-15005)	non-silent gct -> gAt;
NZ_ACHT01000236	10652	T	T	T	T	c	c	c	c	c	c	c	c	c	c	XcampmN_010100007340 metallopeptidase (8644-10743)	non-silent gga -> gAa;
NZ_ACHT01000520	5360	A	A	A	A	g	g	g	g	g	g	g	g	g	g	XcampmN_010100016692 5-methyltetrahydrofolate-homocysteine methyl transferase (5050-618	non-silent ggc -> Agc;
NZ_ACHT01000104	13081	T	T	T	T	g	g	g	g	g	g	g	g	g	g	XcampmN_010100003612 GTP-dependent nucleic acid-binding protein EngD (12772-13863)	non-silent gtg -> Ttg;
NZ_ACHT01000059	1907	C	C	C	C	t	t	t	t	t	t	t	t	t	t	XcampmN_010100001687 putative sugar transporter component (1789-3288)	non-silent gtt -> gCt;
NZ_ACHT01000294	2184	G	G	G	G	t	t	t	t	t	t	t	t	t	t	XcampmN_010100009424 xanthan biosynthesis glucuronosyltransferase GumK (1481-2677)	non-silent tac -> tCc;
NZ_ACHT01000036	320	G	G	G	G	a	a	a	a	a	a	a	a	a	a	XcampmN_010100000817 hypothetical protein (226-321)	non-silent tag -> tGg;
NZ_ACHT01000694	10665	A	A	A	A	t	t	t	t	t	t	t	t	t	t	XcampmN_010100022153 peptide-acetyl-coenzyme A transporter family protein (9657-10952)	non-silent tat -> Aat;
NZ_ACHT01000648	29556	C	C	C	C	a	a	a	a	a	a	a	a	a	a	XcampmN_010100020518 hypothetical protein (28693-29607)	non-silent tat -> Gat;
NZ_ACHT01000720	19485	T	T	T	T	c	c	c	c	c	c	c	c	c	c	XcampmN_010100023003 drug:proton antiporter (19121-20371)	non-silent tcg -> tTg;
NZ_ACHT01000345	1576	T	T	T	T	c	c	c	c	c	c	c	c	c	c	XcampmN_010100010814 cytochrome C peroxidase (1161-2153)	non-silent tgc -> tAc;
NZ_ACHT01000442	4670	G	G	G	G	t	t	t	t	t	t	t	t	t	t	XcampmN_010100013863 arabinogalactan endo-1%2C4-beta-galactosidase (4166-5197)	non-silent ttg -> Gtg;
NZ_ACHT01000104	1160	T	T	T	T	c	c	c	c	c	c	c	c	c	c	XcampmN_010100003552 transcriptional regulator (980-1762)	silent acg -> acA;
NZ_ACHT01000413	9901	C	C	C	C	g	g	g	g	g	g	g	g	g	g	XcampmN_010100012749 ATP-dependent serine activating enzyme (7706-11674)	silent acg -> acC;
NZ_ACHT01000541	32457	G	G	G	G	c	c	c	c	c	c	c	c	c	c	XcampmN_010100017981 23S rRNA 5-methyluridine methyltransferase (31155-32489)	silent acg -> acC;
NZ_ACHT01000083	18782	T	T	T	T	c	c	c	c	c	c	c	c	c	c	XcampmN_010100002657 TonB-dependent receptor (17946-19145)	silent agc -> agT;
NZ_ACHT01000124	5635	C	C	C	C	t	t	t	t	t	t	t	t	t	t	XcampmN_010100004274 urocanate hydratase (5570-6563)	silent agt -> agC;
NZ_ACHT01000552	5493	G	G	G	G	a	a	a	a	a	a	a	a	a	a	XcampmN_010100018498 hypothetical protein (5388-5570)	silent agt -> agC;
NZ_ACHT01000186	13176	A	A	A	A	g	g	g	g	g	g	g	g	g	g	XcampmN_010100005568 hypothetical protein (12886-13461)	silent cag -> caA;
NZ_ACHT01000240	14857	T	T	T	T	c	c	c	c	c	c	c	c	c	c	XcampmN_010100007510 hypothetical protein (14698-15159)	silent cag -> caA;
NZ_ACHT01000683	21403	G	G	G	G	c	c	c	c	c	c	c	c	c	c	XcampmN_010100021898 hypothetical protein (21304-21801)	silent cgg -> cgC;
NZ_ACHT01000202	8056	C	C	C	C	t	t	t	t	t	t	t	t	t	t	XcampmN_010100006263 2-keto-3-deoxygluconate permease (7384-8235)	silent cta -> ctG;
NZ_ACHT01000101	1031	A	A	A	A	g	g	g	g	g	g	g	g	g	g	XcampmN_010100003517 soluble lytic murein transglycosylase (1-1051)	silent ctc -> ctT;
NZ_ACHT01000294	23952	A	A	A	A	g	g	g	g	g	g	g	g	g	g	XcampmN_010100009524 hypothetical protein (23280-24041)	silent ctc -> ctT;
NZ_ACHT01000027	4482	T	T	T	T	c	c	c	c	c	c	c	c	c	c	XcampmN_010100000517 ATP-dependent helicase (3714-5738)	silent ctg -> Ttg;
NZ_ACHT01000081	6150	A	A	A	A	g	g	g	g	g	g	g	g	g	g	XcampmN_010100002462 hypothetical protein (6082-6324)	silent ctg -> Ttg;
NZ_ACHT01000521	4664	T	T	T	T	c	c	c	c	c	c	c	c	c	c	XcampmN_010100016824 hypothetical protein (3494-4993)	silent ctg -> Ttg;
NZ_ACHT01000491	11984	G	G	G	G	t	t	t	t	t	t	t	t	t	t	XcampmN_010100015417 hypothetical protein (11850-12095)	silent ctt -> ctG;
NZ_ACHT01000140	2515	T	T	T	T	c	c	c	c	c	c	c	c	c	c	XcampmN_010100004541 hypothetical protein (2339-2650)	silent gac -> gaT;
NZ_ACHT01000140	2530	C	C	C	C	t	t	t	t	t	t	t	t	t	t	XcampmN_010100004541 hypothetical protein (2339-2650)	silent gat -> gaC;
NZ_ACHT01000539	9001	A	A	A	A	g	g	g	g	g	g	g	g	g	g	XcampmN_010100017716 hypothetical protein (7691-10255)	silent gcg -> gcA;
NZ_ACHT01000696	11781	A	A	A	A	g	g	g	g	g	g	g	g	g	g	XcampmN_010100022268 NAD-binding domain 4%2C putative (11710-12390)	silent gcg -> gcA;
NZ_ACHT01000086	240	C	C	C	C	a	a	a	a	a	a	a	a	a	a	XcampmN_010100002772 hypothetical protein (1-293)	silent ggt -> ggG;

RefSeq accession	position	2005	2251	4387	4389	4379	4380	4381	4383	4384	4394	4392	4395	4433	4434	genes	silent/non-silent
NZ_ACHT01000004	7459	G	G	G	G	a	a	a	a	a	a	a	a	a	a	XcampmN_010100000045 histone-like nucleoid-structuring protein (7127-7471)	silent ttg -> Ctg;
NZ_ACHT01000229	982	C	C	C	C	t	t	t	t	t	t	t	t	t	t	XcampmN_010100007125 leucyl-tRNA synthetase (76-2718)	silent ttg -> Ctg;
NZ_ACHT01000245	44184	G	G	G	G	a	a	a	a	a	a	a	a	a	a	XcampmN_010100008020 putative flavoprotein-ubiquinone oxidoreductase (43207-44856)	silent ttg -> Ctg;
NZ_ACHT01000308	6394	C	C	C	C	t	t	t	t	t	t	t	t	t	t	XcampmN_010100010087 oxidoreductase (5989-7011)	silent ttg -> Ctg;
NZ_ACHT01000344	7594	G	G	G	G	a	a	a	a	a	a	a	a	a	a	XcampmN_010100010799 hypothetical protein (7058-8008)	silent ttg -> Ctg;
NZ_ACHT01000374	11858	C	C	C	C	t	t	t	t	t	t	t	t	t	t	XcampmN_010100011573 Fis family transcriptional regulator (11777-12145)	silent ttg -> Ctg;
NZ_ACHT01000516	3288	G	G	G	G	a	a	a	a	a	a	a	a	a	a	XcampmN_010100016522 hypothetical protein (3151-3678)	silent ttg -> Ctg;
NZ_ACHT01000045	32848	G	G	G	G	a	a	a	a	a	a	a	a	a	a	Intergenic	Intergenic
NZ_ACHT01000064	737	G	G	G	G	c	c	c	c	c	c	c	c	c	c	Intergenic	Intergenic
NZ_ACHT01000090	2705	A	A	A	A	c	c	c	c	c	c	c	c	c	c	Intergenic	Intergenic
NZ_ACHT01000112	8874	T	T	T	T	g	g	g	g	g	g	g	g	g	g	Intergenic	Intergenic
NZ_ACHT01000159	1692	A	A	A	A	g	g	g	g	g	g	g	g	g	g	Intergenic	Intergenic
NZ_ACHT01000196	16252	G	G	G	G	t	t	t	t	t	t	t	t	t	t	Intergenic	Intergenic
NZ_ACHT01000252	13163	G	G	G	G	t	t	t	t	t	t	t	t	t	t	Intergenic	Intergenic
NZ_ACHT01000304	10330	G	G	G	G	a	a	a	a	a	a	a	a	a	a	Intergenic	Intergenic
NZ_ACHT01000398	4545	G	G	G	G	a	a	a	a	a	a	a	a	a	a	Intergenic	Intergenic
NZ_ACHT01000472	4940	C	C	C	C	t	t	t	t	t	t	t	t	t	t	Intergenic	Intergenic
NZ_ACHT01000515	148	C	C	C	C	g	g	g	g	g	g	g	g	g	g	Intergenic	Intergenic
NZ_ACHT01000541	19346	C	C	C	C	t	t	t	t	t	t	t	t	t	t	Intergenic	Intergenic
NZ_ACHT01000615	2742	T	T	T	T	c	c	c	c	c	c	c	c	c	c	Intergenic	Intergenic
NZ_ACHT01000640	6712	G	G	G	G	t	t	t	t	t	t	t	t	t	t	Intergenic	Intergenic
NZ_ACHT01000666	7146	G	G	G	G	a	a	a	a	a	a	a	a	a	a	Intergenic	Intergenic
NZ_ACHT01000666	8084	T	T	T	T	g	g	g	g	g	g	g	g	g	g	Intergenic	Intergenic
NZ_ACHT01000675	6085	C	C	C	C	a	a	a	a	a	a	a	a	a	a	Intergenic	Intergenic
NZ_ACHT01000694	15629	T	T	T	T	c	c	c	c	c	c	c	c	c	c	Intergenic	Intergenic
NZ_ACHT01000694	15630	C	C	C	C	g	g	g	g	g	g	g	g	g	g	Intergenic	Intergenic
NZ_ACHT01000701	859	G	G	G	G	t	t	t	t	t	t	t	t	t	t	Intergenic	Intergenic
