# Supplementary material for: Genome-Wide Sequencing Reveals Two Major Sub-Lineages in the Genetically Monomorphic Pathogen Xanthomonas Campestris Pathovar Musacearum
Source: Genes (Basel). 2012 Jul 4;3(3):361–77. doi: 10.3390/genes3030361 (PMC3902798; doi:10.3390/genes3030361)
Supplement: Supplementary File 4 — PDF-Document (PDF, 357 KB) [file genes-03-00361-s004.pdf]

seq_id	position	2005	2251	4387	4389	4379	4380	4381	4383	4384	4394	4392	4395	4433	4434	genes	silent/non-silent
NZ_ACHT01000023	16591	G	t	t	t	t	t	t	t	t	t	t	t	t	t	Intergenic	Intergenic
NZ_ACHT01000028	7775	A	c	c	c	c	c	c	c	c	c	c	c	c	c	XcampmN_010100000587 ribosomal protein alanine acetyltransferase (7614-8207)	non-silent tac -> taA;
NZ_ACHT01000041	15615	C	t	t	t	t	t	t	t	t	t	t	t	t	t	XcampmN_010100000977 hemolysin III (15149-15805)	non-silent atc -> aCc;
NZ_ACHT01000044	2934	T	c	c	c	c	c	c	c	c	c	c	c	c	c	XcampmN_010100001152 hypothetical protein (2671-3549)	non-silent gat -> Aat;
NZ_ACHT01000044	3736	A	g	g	g	g	g	g	g	g	g	g	g	g	g	XcampmN_010100001157 bifunctional aspartate kinase/diaminopimelate decarboxylase	non-silent gct -> Act;
NZ_ACHT01000060	1984	C	a	a	a	a	a	a	a	a	a	a	a	a	a	Intergenic	Intergenic
NZ_ACHT01000064	1939	C	t	t	t	t	t	t	t	t	t	t	t	t	t	Intergenic	Intergenic
NZ_ACHT01000072	4507	A	c	c	c	c	c	c	c	c	c	c	c	c	c	XcampmN_010100002109 VirB3 protein (4240-4551)	non-silent atg -> atT;
NZ_ACHT01000089	30693	A	c	c	c	c	c	c	c	c	c	c	c	c	c	XcampmN_010100002947 hypothetical protein (30250-31116)	silent atc -> atA;
NZ_ACHT01000101	1035	T	c	c	c	c	c	c	c	c	c	c	c	c	c	XcampmN_010100003517 soluble lytic murein transglycosylase (1-1051)	non-silent cgc -> cAc;
NZ_ACHT01000101	2893	T	c	c	c	c	c	c	c	c	c	c	c	c	c	XcampmN_010100003522 TonB-dependent receptor (1308-4187)	non-silent gcg -> gTg;
NZ_ACHT01000120	419	A	c	c	c	c	c	c	c	c	c	c	c	c	c	XcampmN_010100004174 acyl carrier protein phosphodiesterase (162-746)	non-silent tac -> taA;
NZ_ACHT01000123	3457	T	c	c	c	c	c	c	c	c	c	c	c	c	c	XcampmN_010100004234 hypothetical protein (3202-3477)	silent ctg -> Ttg;
NZ_ACHT01000175	2617	T	c	c	c	c	c	c	c	c	c	c	c	c	c	XcampmN_010100005213 hypothetical protein (1862-2704)	silent aac -> aaT;
NZ_ACHT01000178	5582	T	g	g	g	g	g	g	g	g	g	g	g	g	g	Intergenic	Intergenic
NZ_ACHT01000186	11109	C	g	g	g	g	g	g	g	g	g	g	g	g	g	XcampmN_010100005558 orotidine 5'-phosphate decarboxylase (10740-11471)	silent acc -> acG;
NZ_ACHT01000196	1762	A	c	c	c	c	c	c	c	c	c	c	c	c	c	Intergenic	Intergenic
NZ_ACHT01000209	297	T	c	c	c	c	c	c	c	c	c	c	c	c	c	XcampmN_010100006463 protocatechuate 3%2C4-dioxygenase beta chain (1-684)	silent tac -> taT;
NZ_ACHT01000215	9600	T	c	c	c	c	c	c	c	c	c	c	c	c	c	XcampmN_010100006680 HpaB protein (9387-9857)	silent gcg -> gcA;
NZ_ACHT01000236	9512	C	t	t	t	t	t	t	t	t	t	t	t	t	t	XcampmN_010100007340 metallopeptidase (8644-10743)	non-silent gaa -> gGa;
NZ_ACHT01000245	41520	A	c	c	c	c	c	c	c	c	c	c	c	c	c	XcampmN_010100008015 hypothetical protein (40362-43199)	silent ggg -> ggT;
NZ_ACHT01000256	3474	A	g	g	g	g	g	g	g	g	g	g	g	g	g	XcampmN_010100008560 hypothetical protein (2322-5207)	non-silent gca -> Aca;
NZ_ACHT01000268	5921	T	c	c	c	c	c	c	c	c	c	c	c	c	c	XcampmN_010100008867 general secretory pathway related protein (5108-6922)	silent cag -> caA;
NZ_ACHT01000284	672	A	g	g	g	g	g	g	g	g	g	g	g	g	g	XcampmN_010100009152 hypothetical protein (445-4803)	non-silent ccg -> Tcg;
NZ_ACHT01000303	7530	A	c	c	c	c	c	c	c	c	c	c	c	c	c	XcampmN_010100009850 histidine kinase/response regulator hybrid protein (7183-8451	non-silent gac -> gaA;
NZ_ACHT01000360	1961	A	g	g	g	g	g	g	g	g	g	g	g	g	g	XcampmN_010100011266 two-component system sensor protein (1630-2210)	non-silent tgg -> tAg;
NZ_ACHT01000361	4094	A	c	c	c	c	c	c	c	c	c	c	c	c	c	XcampmN_010100011278 transcription-related protein (3645-6008)	non-silent gca -> Tca;
NZ_ACHT01000374	12027	T	c	c	c	c	c	c	c	c	c	c	c	c	c	XcampmN_010100011573 Fis family transcriptional regulator (11777-12145)	non-silent gct -> gTt;
NZ_ACHT01000378	1614	T	c	c	c	c	c	c	c	c	c	c	c	c	c	XcampmN_010100011643 conjugal transfer relaxosome component TraJ (1608-1979)	silent gag -> gaA;
NZ_ACHT01000382	21607	A	g	g	g	g	g	g	g	g	g	g	g	g	g	XcampmN_010100011748 3-ketoacyl-(acyl-carrier-protein) reductase (21142-21885)	silent acc -> acT;
NZ_ACHT01000388	5277	T	g	g	g	g	g	g	g	g	g	g	g	g	g	XcampmN_010100011860 AraC family transcriptional regulator (4946-5671)	non-silent ggc -> gTc;
NZ_ACHT01000405	6174	A	g	g	g	g	g	g	g	g	g	g	g	g	g	XcampmN_010100012295 ATP-dependent DNA helicase DinG (4482-6617)	non-silent gcc -> Acc;
NZ_ACHT01000438	15721	A	g	g	g	g	g	g	g	g	g	g	g	g	g	XcampmN_010100013693 transposase IS3/IS911 family protein (15704-15877)	silent cta -> Tta;
NZ_ACHT01000439	5166	C	g	g	g	g	g	g	g	g	g	g	g	g	g	XcampmN_010100013743 ECF subfamily RNA polymerase sigma factor (5037-5567)	non-silent gag -> Cag;
NZ_ACHT01000442	14471	C	t	t	t	t	t	t	t	t	t	t	t	t	t	XcampmN_010100013898 hypothetical protein (14031-14639)	silent tat -> taC;
NZ_ACHT01000445	241	A	g	g	g	g	g	g	g	g	g	g	g	g	g	XcampmN_010100013963 carbonic anhydrase (1-308)	non-silent ccg -> cTg;
NZ_ACHT01000450	1683	G	a	a	a	a	a	a	a	a	a	a	a	a	a	Intergenic	Intergenic
NZ_ACHT01000459	3831	T	c	c	c	c	c	c	c	c	c	c	c	c	c	XcampmN_010100014168 aspartyl-tRNA synthetase (2080-3846)	silent cgc -> cgT;
NZ_ACHT01000468	3898	A	g	g	g	g	g	g	g	g	g	g	g	g	g	XcampmN_010100014507 relaxation protein (3589-4158)	silent ggc -> ggT;
NZ_ACHT01000468	3931	A	c	c	c	c	c	c	c	c	c	c	c	c	c	XcampmN_010100014507 relaxation protein (3589-4158)	silent gtg -> gtT;
NZ_ACHT01000468	3955	T	c	c	c	c	c	c	c	c	c	c	c	c	c	XcampmN_010100014507 relaxation protein (3589-4158)	silent gtg -> gtA;
NZ_ACHT01000468	3962	T	c	c	c	c	c	c	c	c	c	c	c	c	c	XcampmN_010100014507 relaxation protein (3589-4158)	non-silent aga -> aAa;
NZ_ACHT01000468	3975	T	c	c	c	c	c	c	c	c	c	c	c	c	c	XcampmN_010100014507 relaxation protein (3589-4158)	non-silent gac -> Aac;
NZ_ACHT01000479	19146	A	c	c	c	c	c	c	c	c	c	c	c	c	c	XcampmN_010100014832 putative glycosyltransferase (18078-19262)	silent ctg -> ctT;
NZ_ACHT01000480	1808	C	g	g	g	g	g	g	g	g	g	g	g	g	g	XcampmN_010100014847 hypothetical protein (1335-1976)	silent acg -> acC;
NZ_ACHT01000480	7300	T	c	c	c	c	c	c	c	c	c	c	c	c	c	XcampmN_010100014877 hypothetical protein (7267-7509)	silent ctg -> Ttg;
NZ_ACHT01000491	4107	T	g	g	g	g	g	g	g	g	g	g	g	g	g	Intergenic	Intergenic
NZ_ACHT01000491	12761	T	c	c	c	c	c	c	c	c	c	c	c	c	c	Intergenic	Intergenic
NZ_ACHT01000493	3280	A	g	g	g	g	g	g	g	g	g	g	g	g	g	XcampmN_010100015452 hypothetical protein (2929-3501)	non-silent ggt -> Agt;
NZ_ACHT01000495	13033	A	g	g	g	g	g	g	g	g	g	g	g	g	g	XcampmN_010100015547 superoxide dismutase (12641-13138)	silent gtg -> gtA;
NZ_ACHT01000508	35194	T	c	c	c	c	c	c	c	c	c	c	c	c	c	XcampmN_010100016342 disulphide-isomerase (34751-36331)	silent aac -> aaT;
NZ_ACHT01000515	1945	A	g	g	g	g	g	g	g	g	g	g	g	g	g	XcampmN_010100016502 23S rRNA m(2)G2445 methyltransferase (1322-2739)	silent gcc -> gcT;
NZ_ACHT01000520	3959	T	c	c	c	c	c	c	c	c	c	c	c	c	c	Intergenic	Intergenic
NZ_ACHT01000539	13258	A	g	g	g	g	g	g	g	g	g	g	g	g	g	XcampmN_010100017736 2'-5' RNA ligase (13049-13651)	silent ctg -> Ttg;
NZ_ACHT01000541	14901	T	c	c	c	c	c	c	c	c	c	c	c	c	c	XcampmN_010100017896 putative sensor protein (14658-15320)	silent ctg -> Ttg;
NZ_ACHT01000550	11623	T	c	c	c	c	c	c	c	c	c	c	c	c	c	XcampmN_010100018361 HrpG (11458-12249)	silent ctg -> Ttg;
NZ_ACHT01000560	2783	C	t	t	t	t	t	t	t	t	t	t	t	t	t	XcampmN_010100018663 molybdopterin biosynthesis (2151-3404)	non-silent agc -> Ggc;

seq_id	position	2005	2251	4387	4389	4379	4380	4381	4383	4384	4394	4392	4395	4433	4434	genes	silent/non-silent
NZ_ACHT01000642	197	A	c	c	c	c	c	c	c	c	c	c	c	c	c	XcampmN_010100020088 hypothetical protein (37-675)	non-silent ggc -> gTc;
NZ_ACHT01000666	5161	C	g	g	g	g	g	g	g	g	g	g	g	g	g	XcampmN_010100021328 hypothetical protein (4518-5378)	non-silent acc -> aGc;
NZ_ACHT01000666	5784	C	g	g	g	g	g	g	g	g	g	g	g	g	g	Intergenic	Intergenic
NZ_ACHT01000668	1036	C	a	a	a	a	a	a	a	a	a	a	a	a	a	XcampmN_010100021383 ABC transporter permease (1-1466)	non-silent ata -> Cta;
NZ_ACHT01000683	20156	T	g	g	g	g	g	g	g	g	g	g	g	g	g	Intergenic	Intergenic
NZ_ACHT01000690	6284	T	g	g	g	g	g	g	g	g	g	g	g	g	g	XcampmN_010100022008 isocitrate dehydrogenase%2C NADP-dependent (5354-7585	non-silent gac -> Tac;
NZ_ACHT01000694	8404	T	g	g	g	g	g	g	g	g	g	g	g	g	g	XcampmN_010100022138 orotate phosphoribosyltransferase (7756-8415)	non-silent cac -> caA;
NZ_ACHT01000713	19319	G	c	c	c	c	c	c	c	c	c	c	c	c	c	XcampmN_010100022678 putative carbon-nitrogen hyrolase family protein (19176-2006	non-silent aac -> aaG;
NZ_ACHT01000713	36931	A	c	c	c	c	c	c	c	c	c	c	c	c	c	XcampmN_010100022743 segregation and condensation protein A (36554-37459)	silent ggc -> ggA;
NZ_ACHT01000733	5016	T	c	c	c	c	c	c	c	c	c	c	c	c	c	XcampmN_010100023330 hypothetical protein (4768-6317)	silent gcg -> gcA;
NZ_ACHT01000004	7459	G	G	G	G	a	a	a	a	a	a	a	a	a	a	XcampmN_010100000045 histone-like nucleoid-structuring protein (7127-7471)	silent ttg -> Ctg;
NZ_ACHT01000013	861	G	G	G	G	c	c	c	c	c	c	c	c	c	c	XcampmN_010100000120 putative ISXo8 transposase (24-929)	non-silent cat -> Gat;
NZ_ACHT01000014	6000	A	A	A	A	g	g	g	g	g	g	g	g	g	g	XcampmN_010100000165 putative monovalent cation/H+ antiporter subunit A (4687-75	non-silent cgg -> Tgg;
NZ_ACHT01000027	4482	T	T	T	T	c	c	c	c	c	c	c	c	c	c	XcampmN_010100000517 ATP-dependent helicase (3714-5738)	silent ctg -> Ttg;
NZ_ACHT01000034	8898	T	T	T	T	c	c	c	c	c	c	c	c	c	c	XcampmN_010100000807 putative integrase protein (8798-8935)	non-silent gca -> gTa;
NZ_ACHT01000036	320	G	G	G	G	a	a	a	a	a	a	a	a	a	a	XcampmN_010100000817 hypothetical protein (226-321)	non-silent tag -> tGg;
NZ_ACHT01000045	1261	A	A	A	A	g	g	g	g	g	g	g	g	g	g	XcampmN_010100001162 bifunctional aspartate kinase/diaminopimelate decarboxylase	non-silent gcg -> Acg;
NZ_ACHT01000045	19682	G	G	G	G	a	a	a	a	a	a	a	a	a	a	XcampmN_010100001247 hypothetical protein (18823-20073)	non-silent aac -> aGc;
NZ_ACHT01000045	32848	G	G	G	G	a	a	a	a	a	a	a	a	a	a	Intergenic	Intergenic
NZ_ACHT01000045	45548	A	A	A	A	c	c	c	c	c	c	c	c	c	c	XcampmN_010100001377 chemotaxis protein (45292-45807)	non-silent agc -> aTc;
NZ_ACHT01000059	1907	C	C	C	C	t	t	t	t	t	t	t	t	t	t	XcampmN_010100001687 putative sugar transporter component (1789-3288)	non-silent gtt -> gCt;
NZ_ACHT01000064	737	G	G	G	G	c	c	c	c	c	c	c	c	c	c	Intergenic	Intergenic
NZ_ACHT01000081	6150	A	A	A	A	g	g	g	g	g	g	g	g	g	g	XcampmN_010100002462 hypothetical protein (6082-6324)	silent ctg -> Ttg;
NZ_ACHT01000083	18782	T	T	T	T	c	c	c	c	c	c	c	c	c	c	XcampmN_010100002657 TonB-dependent receptor (17946-19145)	silent agc -> agT;
NZ_ACHT01000086	240	C	C	C	C	a	a	a	a	a	a	a	a	a	a	XcampmN_010100002772 hypothetical protein (1-293)	silent ggt -> ggG;
NZ_ACHT01000090	2705	A	A	A	A	c	c	c	c	c	c	c	c	c	c	Intergenic	Intergenic
NZ_ACHT01000101	995	G	G	G	G	t	t	t	t	t	t	t	t	t	t	XcampmN_010100003517 soluble lytic murein transglycosylase (1-1051)	non-silent aga -> agC;
NZ_ACHT01000101	1031	A	A	A	A	g	g	g	g	g	g	g	g	g	g	XcampmN_010100003517 soluble lytic murein transglycosylase (1-1051)	silent ctc -> ctT;
NZ_ACHT01000104	1160	T	T	T	T	c	c	c	c	c	c	c	c	c	c	XcampmN_010100003552 transcriptional regulator (980-1762)	silent acg -> acA;
NZ_ACHT01000104	13081	T	T	T	T	g	g	g	g	g	g	g	g	g	g	XcampmN_010100003612 GTP-dependent nucleic acid-binding protein EngD (12772-13	non-silent gtg -> Ttg;
NZ_ACHT01000112	8874	T	T	T	T	g	g	g	g	g	g	g	g	g	g	Intergenic	Intergenic
NZ_ACHT01000113	10410	T	T	T	T	g	g	g	g	g	g	g	g	g	g	XcampmN_010100004062 acetyltransferase (GNAT) family protein (10166-10723)	non-silent gcc -> gAc;
NZ_ACHT01000124	5635	C	C	C	C	t	t	t	t	t	t	t	t	t	t	XcampmN_010100004274 urocanate hydratase (5570-6563)	silent agt -> agC;
NZ_ACHT01000140	2515	T	T	T	T	c	c	c	c	c	c	c	c	c	c	XcampmN_010100004541 hypothetical protein (2339-2650)	silent gac -> gaT;
NZ_ACHT01000140	2530	C	C	C	C	t	t	t	t	t	t	t	t	t	t	XcampmN_010100004541 hypothetical protein (2339-2650)	silent gat -> gaC;
NZ_ACHT01000159	1692	A	A	A	A	g	g	g	g	g	g	g	g	g	g	Intergenic	Intergenic
NZ_ACHT01000175	16953	G	G	G	G	c	c	c	c	c	c	c	c	c	c	XcampmN_010100005303 hypothetical protein (16770-16985)	non-silent ctc -> Gtc;
NZ_ACHT01000186	13176	A	A	A	A	g	g	g	g	g	g	g	g	g	g	XcampmN_010100005568 hypothetical protein (12886-13461)	silent cag -> caA;
NZ_ACHT01000196	16252	G	G	G	G	t	t	t	t	t	t	t	t	t	t	Intergenic	Intergenic
NZ_ACHT01000202	8056	C	C	C	C	t	t	t	t	t	t	t	t	t	t	XcampmN_010100006263 2-keto-3-deoxygluconate permease (7384-8235)	silent cta -> ctG;
NZ_ACHT01000229	982	C	C	C	C	t	t	t	t	t	t	t	t	t	t	XcampmN_010100007125 leucyl-tRNA synthetase (76-2718)	silent ttg -> Ctg;
NZ_ACHT01000236	10652	T	T	T	T	c	c	c	c	c	c	c	c	c	c	XcampmN_010100007340 metallopeptidase (8644-10743)	non-silent gga -> gAa;
NZ_ACHT01000240	14857	T	T	T	T	c	c	c	c	c	c	c	c	c	c	XcampmN_010100007510 hypothetical protein (14698-15159)	silent cag -> caA;
NZ_ACHT01000242	10465	A	A	A	A	c	c	c	c	c	c	c	c	c	c	XcampmN_010100007585 dihydrolipoamide acetyltransferase (8913-10673)	non-silent acg -> aAg;
NZ_ACHT01000245	44184	G	G	G	G	a	a	a	a	a	a	a	a	a	a	XcampmN_010100008020 putative flavoprotein-ubiquinone oxidoreductase (43207-4485	silent ttg -> Ctg;
NZ_ACHT01000252	13163	G	G	G	G	t	t	t	t	t	t	t	t	t	t	Intergenic	Intergenic
NZ_ACHT01000267	349	T	T	T	T	c	c	c	c	c	c	c	c	c	c	XcampmN_010100008857 hypothetical protein (9-1705)	non-silent gcc -> gTc;
NZ_ACHT01000268	14452	T	T	T	T	g	g	g	g	g	g	g	g	g	g	XcampmN_010100008887 hypothetical protein (12246-15005)	non-silent gct -> gAt;
NZ_ACHT01000284	4251	C	C	C	C	t	t	t	t	t	t	t	t	t	t	XcampmN_010100009152 hypothetical protein (445-4803)	non-silent aca -> Gca;
NZ_ACHT01000294	2184	G	G	G	G	t	t	t	t	t	t	t	t	t	t	XcampmN_010100009424 xanthan biosynthesis glucuronosyltransferase GumK (1481-2	non-silent tac -> tCc;
NZ_ACHT01000294	23952	A	A	A	A	g	g	g	g	g	g	g	g	g	g	XcampmN_010100009524 hypothetical protein (23280-24041)	silent ctc -> ctT;
NZ_ACHT01000296	6204	C	C	C	C	t	t	t	t	t	t	t	t	t	t	XcampmN_010100009626 hypothetical protein (5581-6774)	non-silent atg -> Gtg;
NZ_ACHT01000304	10330	G	G	G	G	a	a	a	a	a	a	a	a	a	a	Intergenic	Intergenic
NZ_ACHT01000308	6394	C	C	C	C	t	t	t	t	t	t	t	t	t	t	XcampmN_010100010087 oxidoreductase (5989-7011)	silent ttg -> Ctg;
NZ_ACHT01000344	7594	G	G	G	G	a	a	a	a	a	a	a	a	a	a	XcampmN_010100010799 hypothetical protein (7058-8008)	silent ttg -> Ctg;

seq_id	position	2005	2251	4387	4389	4379	4380	4381	4383	4384	4394	4392	4395	4433	4434	genes	silent/non-silent
NZ_ACHT01000345	1576	T	T	T	T	c	c	c	c	c	c	c	c	c	c	XcampmN_010100010814 cytochrome C peroxidase (1161-2153)	non-silent tgc -> tAc;
NZ_ACHT01000374	11858	C	C	C	C	t	t	t	t	t	t	t	t	t	t	XcampmN_010100011573 Fis family transcriptional regulator (11777-12145)	silent ttg -> Ctg;
NZ_ACHT01000388	4711	T	T	T	T	g	g	g	g	g	g	g	g	g	g	XcampmN_010100011855 hypothetical protein (4350-4814)	non-silent cgt -> cTt;
NZ_ACHT01000398	4545	G	G	G	G	a	a	a	a	a	a	a	a	a	a	Intergenic	Intergenic
NZ_ACHT01000402	4858	T	T	T	T	c	c	c	c	c	c	c	c	c	c	XcampmN_010100012145 heavy metal transporter (4406-5386)	non-silent gat -> Aat;
NZ_ACHT01000404	632	G	G	G	G	a	a	a	a	a	a	a	a	a	a	XcampmN_010100012200 tryptophan halogenase (622-2175)	non-silent ctt -> cCt;
NZ_ACHT01000407	2050	G	G	G	G	t	t	t	t	t	t	t	t	t	t	XcampmN_010100012345 hypothetical protein (1446-3047)	non-silent ctc -> cGc;
NZ_ACHT01000413	9901	C	C	C	C	g	g	g	g	g	g	g	g	g	g	XcampmN_010100012749 ATP-dependent serine activating enzyme (7706-11674)	silent acg -> acC;
NZ_ACHT01000442	4670	G	G	G	G	t	t	t	t	t	t	t	t	t	t	XcampmN_010100013863 arabinogalactan endo-1%2C4-beta-galactosidase (4166-519	non-silent ttg -> Gtg;
NZ_ACHT01000472	4940	C	C	C	C	t	t	t	t	t	t	t	t	t	t	Intergenic	Intergenic
NZ_ACHT01000474	4733	G	G	G	G	a	a	a	a	a	a	a	a	a	a	XcampmN_010100014587 putative secreted protein (3265-5007)	non-silent gac -> gGc;
NZ_ACHT01000491	11984	G	G	G	G	t	t	t	t	t	t	t	t	t	t	XcampmN_010100015417 hypothetical protein (11850-12095)	silent ctt -> ctG;
NZ_ACHT01000500	23584	A	A	A	A	g	g	g	g	g	g	g	g	g	g	XcampmN_010100016057 putative polysaccharide deacetylase (23526-24416)	non-silent gcc -> gTc;
NZ_ACHT01000515	148	C	C	C	C	g	g	g	g	g	g	g	g	g	g	Intergenic	Intergenic
NZ_ACHT01000516	3288	G	G	G	G	a	a	a	a	a	a	a	a	a	a	XcampmN_010100016522 hypothetical protein (3151-3678)	silent ttg -> Ctg;
NZ_ACHT01000520	5360	A	A	A	A	g	g	g	g	g	g	g	g	g	g	XcampmN_010100016692 5-methyltetrahydrofolate-homocysteine methyl transferase (5	non-silent ggc -> gAc;
NZ_ACHT01000521	4664	T	T	T	T	c	c	c	c	c	c	c	c	c	c	XcampmN_010100016824 hypothetical protein (3494-4993)	silent ctg -> Ttg;
NZ_ACHT01000539	9001	A	A	A	A	g	g	g	g	g	g	g	g	g	g	XcampmN_010100017716 hypothetical protein (7691-10255)	silent gcg -> gcA;
NZ_ACHT01000541	19346	C	C	C	C	t	t	t	t	t	t	t	t	t	t	Intergenic	Intergenic
NZ_ACHT01000541	32457	G	G	G	G	c	c	c	c	c	c	c	c	c	c	XcampmN_010100017981 23S rRNA 5-methyluridine methyltransferase (31155-32489)	silent acg -> acC;
NZ_ACHT01000549	7371	A	A	A	A	c	c	c	c	c	c	c	c	c	c	XcampmN_010100018271 two-component system sensor protein (6453-9626)	non-silent cgc -> Agc;
NZ_ACHT01000552	5493	G	G	G	G	a	a	a	a	a	a	a	a	a	a	XcampmN_010100018498 hypothetical protein (5388-5570)	silent agt -> agC;
NZ_ACHT01000560	4001	T	T	T	T	c	c	c	c	c	c	c	c	c	c	XcampmN_010100018673 exodeoxyribonuclease III (3924-4913)	non-silent gct -> Act;
NZ_ACHT01000590	927	C	C	C	C	t	t	t	t	t	t	t	t	t	t	XcampmN_010100019303 RNA polymerase sigma factor (518-1285)	non-silent ctc -> cCc;
NZ_ACHT01000615	2742	T	T	T	T	c	c	c	c	c	c	c	c	c	c	Intergenic	Intergenic
NZ_ACHT01000626	10220	T	T	T	T	c	c	c	c	c	c	c	c	c	c	XcampmN_010100019733 putative glutathionylspermidine synthase (9136-10302)	non-silent cct -> cTt;
NZ_ACHT01000634	2345	T	T	T	T	c	c	c	c	c	c	c	c	c	c	XcampmN_010100019848 beta-mannosidase precursor (2314-3761)	non-silent gcc -> gTc;
NZ_ACHT01000640	6712	G	G	G	G	t	t	t	t	t	t	t	t	t	t	Intergenic	Intergenic
NZ_ACHT01000644	2590	G	G	G	G	a	a	a	a	a	a	a	a	a	a	XcampmN_010100020168 two-component system sensor protein (1-2672)	non-silent act -> Gct;
NZ_ACHT01000648	29556	C	C	C	C	a	a	a	a	a	a	a	a	a	a	XcampmN_010100020518 hypothetical protein (28693-29607)	non-silent tat -> Gat;
NZ_ACHT01000666	7146	G	G	G	G	a	a	a	a	a	a	a	a	a	a	Intergenic	Intergenic
NZ_ACHT01000666	8084	T	T	T	T	g	g	g	g	g	g	g	g	g	g	Intergenic	Intergenic
NZ_ACHT01000675	6085	C	C	C	C	a	a	a	a	a	a	a	a	a	a	Intergenic	Intergenic
NZ_ACHT01000683	21403	G	G	G	G	c	c	c	c	c	c	c	c	c	c	XcampmN_010100021898 hypothetical protein (21304-21801)	silent cgg -> cgC;
NZ_ACHT01000689	3576	A	A	A	A	g	g	g	g	g	g	g	g	g	g	XcampmN_010100021983 crispr-associated protein%2C Csd1 family (3517-5361)	non-silent ctc -> Ttc;
NZ_ACHT01000694	10665	A	A	A	A	t	t	t	t	t	t	t	t	t	t	XcampmN_010100022153 peptide-acetyl-coenzyme A transporter family protein (9657-1	non-silent tat -> Aat;
NZ_ACHT01000694	15629	T	T	T	T	c	c	c	c	c	c	c	c	c	c	Intergenic	Intergenic
NZ_ACHT01000694	15630	C	C	C	C	g	g	g	g	g	g	g	g	g	g		
NZ_ACHT01000696	11781	A	A	A	A	g	g	g	g	g	g	g	g	g	g	XcampmN_010100022268 NAD-binding domain 4%2C putative (11710-12390)	silent gcg -> gcA;
NZ_ACHT01000701	859	G	G	G	G	t	t	t	t	t	t	t	t	t	t		
NZ_ACHT01000720	19485	T	T	T	T	c	c	c	c	c	c	c	c	c	c	XcampmN_010100023003 drug:proton antiporter (19121-20371)	non-silent tcg -> tTg;
NZ_ACHT01000346	5977	A	c	c	c	c	c	c	c	c	c	A	A	c	c	XcampmN_010100010944 hypothetical protein (5604-6218)	non-silent tct -> tAt;
NZ_ACHT01000089	17864	g	A	g	g	g	g	g	g	g	g	g	g	g	g	Intergenic	Intergenic
NZ_ACHT01000104	11913	t	G	t	t	t	t	t	t	t	t	t	t	t	t	XcampmN_010100003602 50S ribosomal protein L25/general stress protein Ctc (11429-	non-silent ttg -> tGg;
NZ_ACHT01000104	28352	a	G	a	a	a	a	a	a	a	a	a	a	a	a	XcampmN_010100003657 DNA-directed RNA polymerase subunit beta' (24193-28410)	non-silent aag -> aGg;
NZ_ACHT01000140	2400	c	A	c	c	c	c	c	c	c	c	c	c	c	c	XcampmN_010100004541 hypothetical protein (2339-2650)	non-silent act -> aAt;
NZ_ACHT01000199	8012	g	T	g	g	g	g	g	g	g	g	g	g	g	g	XcampmN_010100006143 type III secreted effector hopPmaA (6887-8158)	non-silent ttc -> ttA;
NZ_ACHT01000215	3229	c	T	c	c	c	c	c	c	c	c	c	c	c	c	XcampmN_010100006660 HrpF protein (2601-5183)	non-silent ggg -> gAg;
NZ_ACHT01000223	8945	c	T	c	c	c	c	c	c	c	c	c	c	c	c	XcampmN_010100006945 RNA polymerase ECF-type sigma factor (8585-9022)	silent ctg -> ctA;
NZ_ACHT01000499	17684	c	T	c	c	c	c	c	c	c	c	c	c	c	c	Intergenic	Intergenic
NZ_ACHT01000551	5522	c	T	c	c	c	c	c	c	c	c	c	c	c	c	XcampmN_010100018453 excinuclease ABC subunit A (4595-6156)	non-silent cgc -> Tgc;
NZ_ACHT01000609	10047	g	A	g	g	g	g	g	g	g	g	g	g	g	g	Intergenic	Intergenic
NZ_ACHT01000666	4108	c	T	c	c	c	c	c	c	c	c	c	c	c	c	XcampmN_010100021323 hypothetical protein (4027-4458)	silent gag -> gaA;

seq_id	position	2005	2251	4387	4389	4379	4380	4381	4383	4384	4394	4392	4395	4433	4434	genes	silent/non-silent
NZ_ACHT01000721	1937	c	T	c	c	c	c	c	c	c	c	c	c	c	c	XcampmN_010100023025 dihydroorotase (896-2245)	silent ctg -> Ttg;
NZ_ACHT01000726	10603	a	G	a	a	a	a	a	a	a	a	a	a	a	a	Intergenic	Intergenic
NZ_ACHT01000033	777	c	A	A	A	c	c	c	c	c	c	c	c	c	c	XcampmN_010100000737 hypothetical protein (463-1539)	non-silent gct -> Tct;
NZ_ACHT01000043	10914	c	T	T	T	c	c	c	c	c	c	c	c	c	c	XcampmN_010100001107 putative siderophore biosynthesis protein (9991-11760)	silent agc -> agT;
NZ_ACHT01000045	12533	c	A	A	A	c	c	c	c	c	c	c	c	c	c	XcampmN_010100001212 putative xanthine dehydrogenase iron-sulfur-binding subunit	silent cgc -> cgA;
NZ_ACHT01000045	46634	c	T	T	T	c	c	c	c	c	c	c	c	c	c	XcampmN_010100001382 chemotaxis protein (45818-47971)	silent cag -> caA;
NZ_ACHT01000060	2002	g	C	C	C	g	g	g	g	g	g	g	g	g	g	Intergenic	Intergenic
NZ_ACHT01000080	976	c	T	T	T	c	c	c	c	c	c	c	c	c	c	XcampmN_010100002427 histidine kinase/response regulator hybrid protein (1-2185)	non-silent gac -> Aac;
NZ_ACHT01000089	7071	g	A	A	A	g	g	g	g	g	g	g	g	g	g	XcampmN_010100002867 GGDEF family protein (7068-7727)	silent agc -> agT;
NZ_ACHT01000091	253	c	T	T	T	c	c	c	c	c	c	c	c	c	c	XcampmN_010100003067 putative secreted protein (20-4942)	silent ttc -> ttT;
NZ_ACHT01000091	8390	c	A	A	A	c	c	c	c	c	c	c	c	c	c	Intergenic	Intergenic
NZ_ACHT01000099	23369	c	T	T	T	c	c	c	c	c	c	c	c	c	c	XcampmN_010100003437 N-acetylglucosaminidase (22499-24895)	silent ctg -> Ttg;
NZ_ACHT01000138	21001	c	T	T	T	c	c	c	c	c	c	c	c	c	c	Intergenic	Intergenic
NZ_ACHT01000140	1116	c	T	T	T	c	c	c	c	c	c	c	c	c	c	XcampmN_010100004536 LacI family transcription regulator (1016-2011)	non-silent gcc -> gTc;
NZ_ACHT01000205	131	g	A	A	A	g	g	g	g	g	g	g	g	g	g	XcampmN_010100006318 putative integrase (1-859)	non-silent cgt -> cAt;
NZ_ACHT01000229	981	g	A	A	A	g	g	g	g	g	g	g	g	g	g	XcampmN_010100007125 leucyl-tRNA synthetase (76-2718)	silent gag -> gaA;
NZ_ACHT01000242	12462	g	A	A	A	g	g	g	g	g	g	g	g	g	g	XcampmN_010100007595 dihydrolipoamide dehydrogenase (11334-13157)	non-silent gcc -> Acc;
NZ_ACHT01000250	10340	g	C	C	C	g	g	g	g	g	g	g	g	g	g	XcampmN_010100008245 hypothetical protein (9986-10390)	non-silent ggg -> Cgg;
NZ_ACHT01000256	13371	g	C	C	C	g	g	g	g	g	g	g	g	g	g	XcampmN_010100008595 hypothetical protein (12964-13470)	non-silent ctg -> Gtg;
NZ_ACHT01000294	31553	g	A	A	A	g	g	g	g	g	g	g	g	g	g	XcampmN_010100009559 MFS transporter (30245-31570)	non-silent gcg -> Acg;
NZ_ACHT01000296	5624	a	C	C	C	a	a	a	a	a	a	a	a	a	a	XcampmN_010100009626 hypothetical protein (5581-6774)	non-silent ctg -> cGg;
NZ_ACHT01000332	2191	a	G	G	G	a	a	a	a	a	a	a	a	a	a	XcampmN_010100010574 putative filamentous hemagglutinin-like protein (46-7976)	non-silent aca -> Gca;
NZ_ACHT01000382	10916	g	T	T	T	g	g	g	g	g	g	g	g	g	g	XcampmN_010100011693 2-methylisocitrate lyase (10912-11808)	non-silent gca -> gAa;
NZ_ACHT01000396	3578	c	G	G	G	c	c	c	c	c	c	c	c	c	c	XcampmN_010100011920 catalase (3375-5483)	non-silent gaa -> Caa;
NZ_ACHT01000413	4760	g	A	A	A	g	g	g	g	g	g	g	g	g	g	Intergenic	Intergenic
NZ_ACHT01000418	297	t	G	G	G	t	t	t	t	t	t	t	t	t	t	Intergenic	Intergenic
NZ_ACHT01000420	5559	c	G	G	G	c	c	c	c	c	c	c	c	c	c	XcampmN_010100013024 putative 2OG-Fe(II) oxygenase superfamily protein (4797-57	silent ctg -> ctC;
NZ_ACHT01000421	8792	g	T	T	T	g	g	g	g	g	g	g	g	g	g	Intergenic	Intergenic
NZ_ACHT01000436	3952	a	G	G	G	a	a	a	a	a	a	a	a	a	a	XcampmN_010100013633 XopX effector protein (2588-4771)	silent ttg -> Ctg;
NZ_ACHT01000468	3715	g	C	C	C	g	g	g	g	g	g	g	g	g	g	XcampmN_010100014507 relaxation protein (3589-4158)	silent gcc -> gcG;
NZ_ACHT01000468	3862	a	T	T	T	a	a	a	a	a	a	a	a	a	a	XcampmN_010100014507 relaxation protein (3589-4158)	non-silent gat -> gaA;
NZ_ACHT01000478	1287	c	T	T	T	c	c	c	c	c	c	c	c	c	c	XcampmN_010100014662 phage-related tail protein (1111-1320)	non-silent gac -> Aac;
NZ_ACHT01000478	8851	g	A	A	A	g	g	g	g	g	g	g	g	g	g	Intergenic	Intergenic
NZ_ACHT01000482	10486	c	T	T	T	c	c	c	c	c	c	c	c	c	c	XcampmN_010100015002 hypothetical protein (10002-10820)	non-silent tcg -> tTg;
NZ_ACHT01000508	22731	g	A	A	A	g	g	g	g	g	g	g	g	g	g	Intergenic	Intergenic
NZ_ACHT01000510	2480	g	A	A	A	g	g	g	g	g	g	g	g	g	g	XcampmN_010100016452 drug:H+ antiporter-1 family protein (2006-3271)	silent agc -> agT;
NZ_ACHT01000518	4688	g	A	A	A	g	g	g	g	g	g	g	g	g	g	XcampmN_010100016582 sulfate transporter (3671-5140)	silent atc -> atT;
NZ_ACHT01000520	15004	c	A	A	A	c	c	c	c	c	c	c	c	c	c	XcampmN_010100016722 hypothetical protein (14338-15102)	silent ccg -> ccT;
NZ_ACHT01000522	830	c	A	A	A	c	c	c	c	c	c	c	c	c	c	XcampmN_010100016839 2-oxoglutarate dehydrogenase E1 component (738-1080)	silent gtc -> gtA;
NZ_ACHT01000532	743	c	T	T	T	c	c	c	c	c	c	c	c	c	c	XcampmN_010100017284 beta-glucosidase (1-1772)	non-silent gcc -> gTc;
NZ_ACHT01000538	3594	t	C	C	C	t	t	t	t	t	t	t	t	t	t	XcampmN_010100017501 hypothetical protein (2378-3724)	non-silent atc -> aCc;
NZ_ACHT01000538	20186	t	C	C	C	t	t	t	t	t	t	t	t	t	t	Intergenic	Intergenic
NZ_ACHT01000549	1367	g	A	A	A	g	g	g	g	g	g	g	g	g	g	Intergenic	Intergenic
NZ_ACHT01000564	18074	c	T	T	T	c	c	c	c	c	c	c	c	c	c	XcampmN_010100018848 ATP-dependent Clp protease subunit (16745-19027)	silent gag -> gaA;
NZ_ACHT01000580	604	g	A	A	A	g	g	g	g	g	g	g	g	g	g	Intergenic	Intergenic
NZ_ACHT01000640	6733	a	G	G	G	a	a	a	a	a	a	a	a	a	a	Intergenic	Intergenic
NZ_ACHT01000648	23525	a	T	T	T	a	a	a	a	a	a	a	a	a	a	XcampmN_010100020488 DNA polymerase I (21271-24072)	non-silent gac -> gTc;
NZ_ACHT01000661	4425	c	T	T	T	c	c	c	c	c	c	c	c	c	c	XcampmN_010100020913 branched-chain amino acid aminotransferase (4229-5314)	non-silent ggt -> gAt;
NZ_ACHT01000675	9320	c	T	T	T	c	c	c	c	c	c	c	c	c	c	XcampmN_010100021653 cell division protein FtsA (8864-10099)	silent gtg -> gtA;
NZ_ACHT01000719	10514	c	T	T	T	c	c	c	c	c	c	c	c	c	c	Intergenic	Intergenic
NZ_ACHT01000725	3818	c	A	A	A	c	c	c	c	c	c	c	c	c	c	XcampmN_010100023135 Putative signal protein with GGDEF and EAL domains (1971	non-silent gcc -> Tcc;
NZ_ACHT01000013	820	c	c	c	c	c	T	c	c	c	c	c	c	c	c	XcampmN_010100000120 putative ISXo8 transposase (24-929)	non-silent gct -> gTt;
NZ_ACHT01000271	1121	c	c	c	c	c	T	c	c	c	c	c	c	c	c	XcampmN_010100008902 valyl-tRNA synthetase (50-2884)	silent gag -> gaA;

seq_id	position	2005	2251	4387	4389	4379	4380	4381	4383	4384	4394	4392	4395	4433	4434	genes	silent/non-silent
NZ_ACHT01000695	2021	c	c	c	c	c	T	c	c	c	c	c	c	c	c	Intergenic	Intergenic
NZ_ACHT01000188	7128	g	g	g	g	g	g	g	A	A	g	g	g	g	g	XcampmN_010100005643 transcriptional regulator (6892-7869)	silent cag -> caA;
NZ_ACHT01000442	7962	c	c	c	c	c	c	c	T	T	c	c	c	c	c	XcampmN_010100013868 pyruvate dehydrogenase subunit E1 (5709-8396)	silent ctg -> Ttg;
NZ_ACHT01000496	10525	c	c	A	c	c	c	c	c	c	c	c	c	c	c	Intergenic	Intergenic
NZ_ACHT01000060	12324	c	c	T	T	c	c	c	c	c	c	c	c	c	c	XcampmN_010100001757 glucan 1%2C4-beta-glucosidase (11521-14187)	silent ggc -> ggT;
NZ_ACHT01000089	33277	g	g	A	A	g	g	g	g	g	g	g	g	g	g	XcampmN_010100002957 glycosidase (33161-34930)	silent cag -> caA;
NZ_ACHT01000124	5863	c	c	T	T	c	c	c	c	c	c	c	c	c	c	XcampmN_010100004274 urocanate hydratase (5570-6563)	silent cgc -> cgT;
NZ_ACHT01000175	13158	g	g	T	T	g	g	g	g	g	g	g	g	g	g	XcampmN_010100005283 hypothetical protein (12890-13762)	non-silent cgg -> cTg;
NZ_ACHT01000213	4160	g	g	A	A	g	g	g	g	g	g	g	g	g	g	XcampmN_010100006598 ISBmu21 transposase (3787-4227)	non-silent agc -> aAc;
NZ_ACHT01000252	20741	c	c	T	T	c	c	c	c	c	c	c	c	c	c	XcampmN_010100008425 arabinosidase (19721-21415)	silent gtg -> gtA;
NZ_ACHT01000374	2270	g	g	A	A	g	g	g	g	g	g	g	g	g	g	XcampmN_010100011543 ATP-dependent DNA helicase RecG (1347-2522)	non-silent ctc -> Ttc;
NZ_ACHT01000421	11740	g	g	A	A	g	g	g	g	g	g	g	g	g	g	Intergenic	Intergenic
NZ_ACHT01000531	1966	c	c	A	A	c	c	c	c	c	c	c	c	c	c	Intergenic	Intergenic
NZ_ACHT01000542	8776	c	c	T	T	c	c	c	c	c	c	c	c	c	c	XcampmN_010100018041 fatty oxidation complex alpha subunit (7549-9630)	silent ctg -> Ttg;
NZ_ACHT01000560	7631	g	g	C	C	g	g	g	g	g	g	g	g	g	g	XcampmN_010100018688 hypothetical protein (7233-7763)	non-silent ctg -> Gtg;
NZ_ACHT01000588	1635	g	g	A	A	g	g	g	g	g	g	g	g	g	g	Intergenic	Intergenic
NZ_ACHT01000749	2901	c	c	T	T	c	c	c	c	c	c	c	c	c	c	XcampmN_010100023637 uracil-DNA glycosylase (2713-3438)	silent ctc -> ctT;
NZ_ACHT01000726	7785	c	c	c	c	c	c	c	c	c	T	c	c	c	c	XcampmN_010100023190 hypothetical protein (6879-8435)	silent cgg -> cgA;
NZ_ACHT01000074	12788	g	g	g	g	g	g	g	g	g	g	T	T	g	g	XcampmN_010100002318 NADH dehydrogenase subunit N (11435-12898)	non-silent ttc -> ttA;
NZ_ACHT01000090	13622	c	c	c	c	c	c	c	c	c	c	A	A	c	c	XcampmN_010100003052 oxidoreductase (12304-13890)	non-silent gcc -> gAc;
NZ_ACHT01000318	8972	c	c	c	c	c	c	c	c	c	c	T	T	c	c	XcampmN_010100010357 imidazole glycerol-phosphate dehydratase/histidinol phospha	non-silent gtc -> Atc;
NZ_ACHT01000720	16469	g	g	g	g	g	g	g	g	g	g	A	A	g	g	XcampmN_010100022988 hypothetical protein (16378-16872)	non-silent tcg -> tTg;
NZ_ACHT01000108	1587	g	g	g	g	g	g	g	g	g	g	g	g	A	g	Intergenic	Intergenic
NZ_ACHT01000459	7169	c	c	c	c	c	c	c	c	c	c	c	c	c	T	XcampmN_010100014198 Holliday junction DNA helicase RuvA (7088-7672)	silent ctg -> Ttg;
NZ_ACHT01000536	6369	c	c	c	c	c	c	c	c	c	c	c	c	c	T	XcampmN_010100017436 spore Coat Protein U domain family protein (5768-6802)	non-silent tcc -> tTc;
